# A systems biology approach to studying the molecular mechanisms of osteoblastic differentiation under cytokine combination treatment

**DOI:** 10.1038/s41536-017-0009-0

**Published:** 2017-03-10

**Authors:** Hua Tan, Ruoying Chen, Wenyang Li, Weiling Zhao, Yuanyuan Zhang, Yunzhi Yang, Jing Su, Xiaobo Zhou

**Affiliations:** 1 Center for Bioinformatics & Systems Biology, Department of Radiology, Wake Forest University School of Medicine, Winston-Salem, NC 27157 USA; 2 Chongqing Key Laboratory of Oral Diseases and Biomedical Sciences and College of Stomatology, Chongqing Medical University, Chongqing, 400016 China; 3 Institute of Regenerative Medicine, Wake Forest University School of Medicine, Winston-Salem, NC 27157 USA; 4 Department of Orthopedic Surgery, Stanford University, Stanford, CA 94305 USA; 5 Department of Materials Science and Engineering, Stanford University, Stanford, CA 94305 USA; 6 Department of Bioengineering, Stanford University, Stanford, CA 94305 USA; 7 School of Electronics and Information Engineering, Tongji University, Shanghai, 201804 China; 8 College of Biomedical Engineering and Instrument Science, Zhejiang University, Hangzhou, Zhejiang 310058 China

## Abstract

Recent studies revealed that sequential release of bone morphogenetic protein 2 and insulin-like growth factor 1 plays an important role in osteogenic process, suggesting that cytokines bone morphogenetic protein 2 and insulin-like growth factor 1 function in a time-dependent manner. However, the specific molecular mechanisms underlying these observations remained elusive, impeding the elaborate manipulation of cytokine sequential delivery in tissue repair. The aim of this study was to identify the key relevant pathways and processes regulating bone morphogenetic protein 2/insulin-like growth factor 1-mediated osteoblastic differentiation. Based on the microarray and proteomics data, and differentiation/growth status of mouse bone marrow stromal cells, we constructed a multiscale systems model to simulate the bone marrow stromal cells lineage commitment and bone morphogenetic protein 2 and insulin-like growth factor 1-regulated signaling dynamics. The accuracy of our model was validated using a set of independent experimental data. Our study reveals that, treatment of bone marrow stromal cells with bone morphogenetic protein 2 prior to insulin-like growth factor 1 led to the activation of transcription factor Runx2 through TAK1-p38 MAPK and SMAD1/5 signaling pathways and initiated the lineage commitment of bone marrow stromal cells. Delivery of insulin-like growth factor 1 four days after bone morphogenetic protein 2 treatment optimally activated transcription factors osterix and β-catenin through ERK and AKT pathways, which are critical to preosteoblast maturity. Our systems biology approach is expected to provide technical and scientific support in optimizing therapeutic scheme to improve osteogenesis/bone regeneration and other essential biological processes.

## Using computer simulations to optimize therapies

Computer simulations using experimental data could optimize bone regeneration treatments. Xiaobo Zhou of Wake Forest University School of Medicine, Yunzhi Yang of Stanford University in the US and colleagues found that sequentially delivering two cell-signaling molecules to mouse bone marrow stromal cells (BMSCs), with four days in between, led to optimal growth of bone-forming cells in comparison to other delivery timings. They treated mouse BMSCs with ‘bone morphogenetic protein 2’ (BMP-2) and/or ‘insulin-like growth factor 1’ (IGF-1), which induce osteoblast differentiation and proliferation respectively. They then input the resultant microarray and proteomics data into a computer simulation model they constructed to study the cells’ behaviors over variable time scales and with varied biological processes. They found that delivering IGF-1 four days after BMP-2 was optimum for activating critical pathways for bone regeneration.

## Introduction

Bone regeneration is a complex process mediated by a series of biophysical events, including stem cell differentiation, neo-vascularization, and mechanical loading.^1–4^ Many cytokines and growth factors such as BMP-2 (bone morphogenetic protein 2) and IGF-1 (insulin-like growth factor 1) play critical roles in regulating these events.^5^ Growth factors tend to interactively regulate cell differentiation and growth. Yeh *et al*. reported that combination of osteogenic protein-1 (OP1) with either IGF-1 or interleukin-6 enhanced OP1-induced increase in cell proliferation, alkaline phosphatase (ALP) activity and bone nodule formation;^6, 7^ while combined treatment with platelet-derived growth factor and IGF-1 improved the periodontal structure healing of periodontitis-affected teeth.^8^ These studies indicated that cytokine combinations have a synergistic effect on bone healing and tissue regeneration, and hence represent a potential therapeutic strategy for the repair of bone defects.

To exploit the clinical potential of cytokine combination therapy, researchers have developed biomaterials and systems to facilitate multiple-cytokine delivery, and thereby improve osteoblastic differentiation in vitro, which is a critical initial step toward clinical application of cytokine combination therapy to bone regeneration.^9–12^ Particularly, combination of BMP-2 and IGF-1 has been widely employed in dual cytokine treatment. BMP-2 is an osteoinductive factor that can potently induce osteoblast differentiation in vitro and in vivo.^13–15^ BMP-2 has been approved by FDA for clinical use. IGF-1 is a mitogenic factor capable of inducing osteoblast proliferation and growth toward local osseointegration.^16, 17^ We and others revealed that sequential delivery of BMP-2 and IGF-1 significantly enhanced osteoblastic differentiation, when compared to the groups treated with either a single cytokine or simultaneous release of both factors.^9, 10, 18^ In the present work, we further specified that delivery of BMP-2 at day 1 and IGF-1 at day 4 (denoted ‘B1I4’) to mouse bone marrow stromal cells (BMSCs) yielded optimal osteoblast formation in comparison with other temporal combinations. Taken together, delivery timing (temporal order) of BMP-2 and IGF-1 is an important variable for obtaining the best outcome.

Identifying proper delivery timing of dual growth factors is critical for designing optimal cytokine combination therapies, and hence poses obvious biological and clinical significance. However, it is costly and time-consuming to experimentally screen all possible temporal combinations. Hence, we proposed a systems biology approach to address this challenge. This approach combined computational modeling and experimental data to investigate the BMSC lineage process. Under this framework, only a small fraction of treatment conditions needs to be covered by experiments, while the remainder can be simulated and predicted using the computational model trained by the experimental data.

In the present study, we first treated mouse BMSCs (w-20-17) by BMP-2 and/or IGF-1. These growth factors directed the initial BMSCs toward osteoblastic differentiation and lineage commitment. Then we elaborately (at many time points) measured the molecular (gene expression and protein phosphorylation profiling) and cellular dynamics (differentiating activity and osteoblast formation) following growth factor delivery. These experimental data provided a unique opportunity for us to infer bone cell lineage-associated signaling pathways, identify activation status of related transcription factors (TFs), and model cell lineage progression under various treatment conditions.

To achieve these goals, we constructed a multiscale systems model to simulate the BMSC lineage commitment under cytokine treatments at both molecular and cellular levels. The multiscale model integrated our experimental data of various scales to represent a coordinated system. By choosing proper parameters, the output of this model was fitted to the experimental data very well. We also evaluated the significance of involved parameters to model output through global sensitivity analysis. In addition, we validated our model with an independent set of experimental data, and consequently proposed a convincing mechanism to explain the outcomes of combined treatment with specific cytokines. Our finding is expected to provide technical and scientific support in optimizing therapeutic scheme involving sequential delivery of dual (or even multiple cytokines) in bone regeneration and other tissue remodeling processes.

## Results

### The integrated systems biology approach

**Fig. 1:**
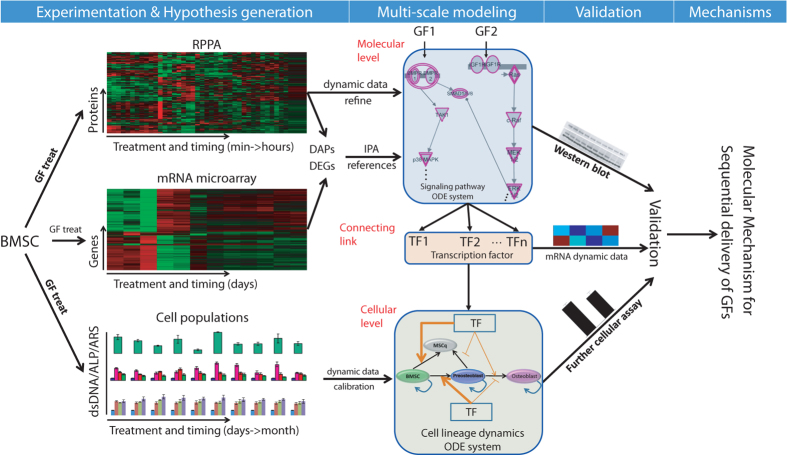
Schematic overview of the systems biology approach. The molecular (mRNA and RPPA) and cellular data (dsDNA, ALP and ARS) are collected experimentally for constructing and calibrating the multiscale model of BMSC lineage commitment. The molecular and cellular components were implemented by separate ODE systems. The model was validated by additional group of experimental data. Term abbreviations are provided in the main text

To understand the underlying molecular mechanisms of bone cell lineage commitment chronologically orchestrated by BMP-2 and IGF-1, we established a systems biology approach to explore the process of bone cell differentiation and growth. As illustrated in Fig. 1, our systems biology scheme consists of four major components: (1) experiment design and data preparation/analysis, (2) multiscale model construction and calibration, (3) model validation with additional experimental data, and (4) mechanistic explanation.

To obtain essential data for model construction, mouse BMSCs (W-20-17) were treated with temporal combinations of BMP-2 and IGF-1 (Table S1), and the cell differentiation and growth were analyzed by measuring dsDNA (double strand DNA) contents (total cell mass), ALP activity (osteoblastic differentiation) and matrix calcium deposition (osteoblast formation). Total RNA and protein samples were collected at various time points for gene expression and protein phospho-signaling analysis using microarray and reverse phase protein array (RPPA) methods, respectively (the *left panel* of Fig. 1). These data were used to construct a multiscale model for simulating cell lineage progression, and the associated signaling transduction was triggered by BMP-2 and IGF-1 (the *middle panel* of Fig. 1). Concretely, dynamic data from RPPA and microarray assays contained important cues regarding the key molecules that may potentially be involved in BMSC differentiation toward osteoblasts. We extracted the differentially activated proteins and differentially expressed genes from these data to generate the generic signaling pathways associated with BMSC differentiation and proliferation (Materials and Methods). The dynamic data of cell population were input into the cellular lineage model for parameter calibration. We implemented the molecular signaling and cellular lineage components with different ordinary differential equation (ODE) systems. The two scales were connected by several critical TFs associated with bone cell differentiation and proliferation. The model was validated by a new set of molecular and cellular data (the *right panel* of Fig. 1).

### Molecular dynamics in comparison with RPPA data

Our molecular ODE system well recapitulated the dynamics of the 12 signaling proteins which were covered by the RPPA data (Supplementary Fig. S1). The parameters used here are listed in Table S2 and Supplementary Fig. S2. The protein profiles manifested obvious difference between treatment groups with single and dual growth factors. Particularly, when the cells were treated with IGF-1 alone (I1), neither p38 MAPK nor PI3K/Akt/mTOR signaling pathways was well activated (Supplementary Fig. S3), and hence all the related downstream TFs Runx2, osterix and β-catenin were expected to be inactivated. This is well consistent with previously reported significance of p38 MAPK pathway in bone homeostasis.^19^ When the cells were treated with BMP-2 first and then IGF-1 (B1I4), ERK, GSK3β, and S6 were phosphorylated (Supplementary Fig. S6). These proteins were the signaling molecules essential for the activation of osterix, β-catenin and cell cycle related proteins. It should be noted that inhibition of Akt on GSK3β, and inhibition of GSK3β on β-catenin, are achieved by phosphorylation, rather than dephosphorylation. This is because the phosphorylated GSK3β and β-catenin will be degraded while their non-phosphorylated part will be active and responsible for downstream stimulation.^20^ This unusual mechanism makes them counterintuitive at first glance, for example, the strong phosphorylation of GSK3β in B1I4 (Supplementary Fig. S6) will actually lead to activation of β-catenin. The molecular dynamics in B1 (Supplementary Fig. S4) shows that both p38 MAPK and SMAD1/5 were phosphorylated, which together lead to the activation of Runx2. Consistently, the p38 MAPK was also activated in I1B4 owing to the immediate BMP-2 stimulation at day 4 (Supplementary Fig. S5). However, its downstream SMAD1/5 was inhibited due to the early inhibitory effect of IGF-1 on ERK at day 1, demonstrating the essential role of ERK in cell cycle regulation.^21^

Our analysis indicated that treatment of BMSCs with temporal combinations of BMP-2 and IGF-1 induced distinct signaling profiles. The signaling activated by one cytokine could be enhanced, curtailed or inactivated by the second cytokine. This is also exemplified in the parameter values (corresponding to phosphorylation and dephosphorylation rates) estimated from different treatment scenarios (Supplementary Fig. S2). There are more parameters with values <0.02 or >0.08 in the I1B4 and B1I4 treatment groups than those in I1 and B1 groups. An unusually small or large coefficient indicates that the signaling transduction is substantially enhanced or impeded in that particular treatment scenario, in comparison with the evenly distributed parameter values. In the B1I4 scenario, the parameters involved in the IGF-1-mediated sub-pathways varied dramatically between each other, reflecting the strong phosphorylation of ERK (or MAPK), GSK3β, and S6 proteins stimulated by IGF-1 (Supplementary Fig. S1).

### Cellular dynamics in comparison with experimental results

**Fig. 2:**
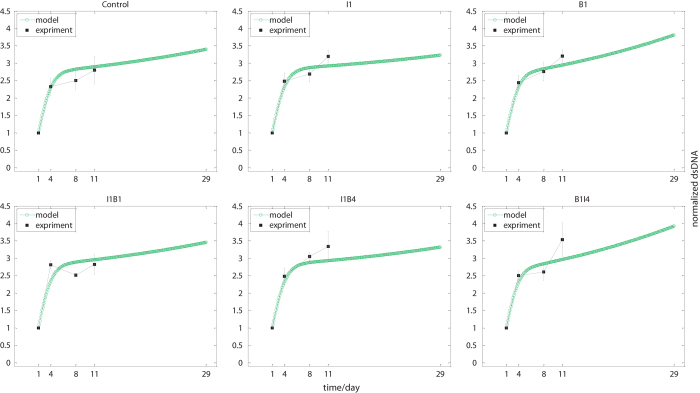
Model simulation and experiment results of cell mass dynamics for various treatments. Experimental and model simulation results are illustrated by *solid squares* and *hollow circles*, respectively. Total cell mass was determined by dsDNA at day 1, 4, 8, 11 post treatment with cytokines. Cell mass were normalized to initial dsDNA mass at day 1. Experimental data are shown as mean ±  SD, *n *= 4, *see* Supplementary Fig. S9B for original dsDNA results

Our cell lineage model successfully reproduced the observations at the cellular level (Fig. 2). The major parameters involved in the cellular lineage model are summarized in Supplementary Table S3. No significant difference in cell growth was seen across all groups at the early stage (during the first 11 days) following growth factor treatment. However, cell differentiation was markedly affected by temporal combination of BMP-2 and IGF-1 from the beginning, as manifested by distinct cellular composition (i.e., the percentage of BMSCs, non-osteoblastic cells and osteoblasts) (Supplementary Fig. S8). The heterogeneity in cellular composition was attributed to the varying activation of essential TFs triggered by different combinations of growth factors. Our study further suggested that different treatment conditions resulted in dramatic variation in final osteoblast formation (Supplementary Fig. S8).

### Synergistic effect of BMP-2 and IGF-1 on BMSC lineage progression

To evaluate the synergistic role of BMP-2 and IGF-1 in osteoblast formation, we conducted a series of experiments to assess the effect of temporal combination of BMP-2 and IGF-1 on BMSC lineage progression (Table S1). Among the six treatment groups, the best results were obtained in the B1I4 treatment scenario in terms of osteoblast formation (determined by ARS), followed by I1B1 and B1, while other conditions (I1B4, I1, control) ranked at the bottom (Supplementary Fig. S8). These results were consistent with the change in ALP activity (Supplementary Fig. S9A in W-20-17 cells and Supplementary Fig. S12 in MC-3T3 cells).

**Fig. 3:**
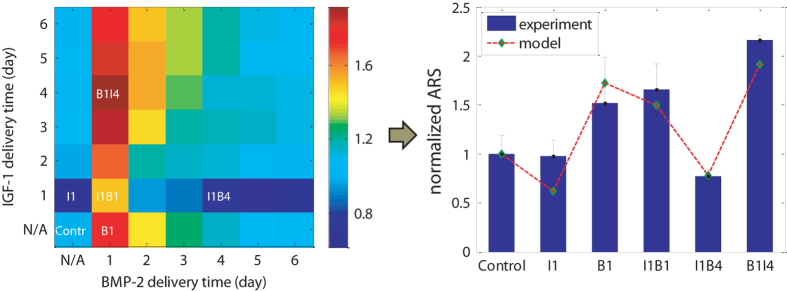
Model prediction and experiment results of osteoblast formation for various treatments. *Left*: The bone cell lineage progression was simulated under 49 possible cytokine combinations with time interval ≤5 days between two sequential deliveries. The treatment conditions covered by experiments are denoted in the corresponding grids. *Right*: Model prediction was compared to experimental data, where osteoblasts production was assessed by matrix calcium deposition measured by ARS staining at day 28 of culture and normalized to control condition (mean ±  SD, *n*=4), *see* Supplementary Fig. S9C for original ARS results

Besides recapitulating the cellular dynamics observed in our experiments, our systems biology approach also presented the ability to screen cytokine combinations, which were not covered by experiments. We conducted a comprehensive in silico ‘grid’ search of possible combinations of BMP-2 and IGF-1 with time intervals ranging from 0 to 5 days between two sequential deliveries, and evaluated their effects on cell lineage progression. Figure 3 illustrates the profiles of osteoblast formation at day 29 for 49 different temporal combinations of BMP-2 and IGF-1. The six treatment scenarios covered by our experiments are denoted in the corresponding grids (the *left panel* of Fig. 3) and compared with predicted results by the model (the *right panel* of Fig. 3). In general, when IGF-1 was added alone or prior to BMP-2, the rate of osteoblast formation was low and even lower if BMP-2 was added after a longer interval. On the contrary, when BMP-2 was added alone or prior to IGF-1, a good yield of osteoblast formation could be achieved. These observations were consistent with the previously reported individual effect of BMP-2 and IGF-1.^22^ In addition, the osteoblast formation was not a monotonic function of the time intervals, and a peak was reached when the time interval was 3 days (B1I4). To conclude, our results indicated that BMP-2 should be delivered prior to IGF-1, and the specific timing for sequential delivery is also critical for optimal osteoblast formation.

### Global sensitivity analysis on model parameters

Our multiscale model involved a batch of parameters. Although most parameters were obtained through fitting to the experimental data, they still potentially included some uncertainty. To address this issue, we performed a global sensitivity analysis on the molecular and cellular model parameters, respectively. The major advantage of global sensitivity analysis compared to regular local method is its ability to assess the overall influence of each individual parameter when all parameters are concurrently perturbed.^23^

**Fig. 4:**
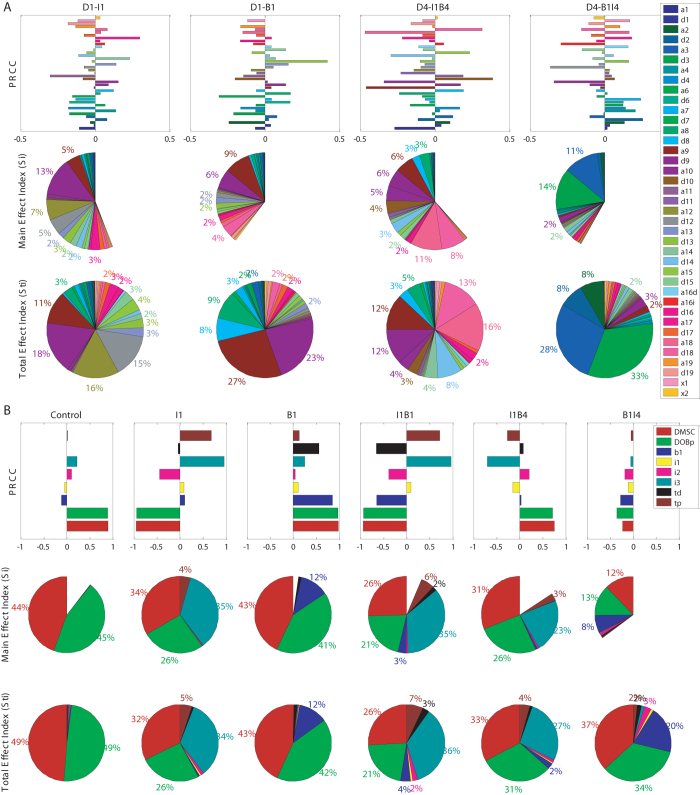
Global sensitivity analysis on the parameters involved in the multiscale model. **a** Global sensitivity analysis on the parameters involved in the molecular ODE system (*see* Supplementary Figure S1 and Table S2 for parameter detail). The analyses were performed separately for four treatments with RPPA data available. **b** Global sensitivity analysis on the parameters involved in the cellular ODE system (*see* Table S3 for parameter detail). The analyses were conducted, respectively, for six treatment conditions with cellular assay data available. *PRCC*: Spearman’s partial rank correlation coefficient; main and total effect index refer to the first order and higher order of sensitivity index calculated by extended Fourier Amplitude Sensitivity Test (eFAST). Only those parameters that account for at least 2% of the output variation are illustrated on the pie charts

Figure 4 shows partial rank correlation coefficients (PRCC) and main/total effect index, indicative of influential parameter perturbations under various treatments. For the molecular ODE system (Fig. 4a), the outcome of B1I4 was most sensitive to p38 MAPK phosphorylation profile (parameters *a*_*3*_ and *d*_*3*_ accounted for 11 and 14% of the output variation respectively) based on the main effect index (the middle panel of Fig. 4a), substantially different from other treatment conditions. Groups I1, B1, and I1B4 were largely determined by the c-Raf signaling transduction (parameters *a*_9_ and *d*_9_ accounted for 5–13% of output variation, *see* Supplementary Fig. S1 for detail of the parameters). These three groups can be further distinguished by the higher-order sensitivity index, i.e., the total effect index (the *lower panel* of Fig. 4a). Particularly, besides c-Raf, I1, B1, and I1B4 are also dominated by PDK1 (*a*_12_ and *d*_12_), Ras (*a*_8_ and *d*_8_), and p70S6K (*a*_18_ and *d*_18_) signaling molecules, respectively. On the other hand, B1I4 is sensitive to TAK1 (*a*_2_ and *d*_2_) in addition to p38 MAPK. Perturbation on other parameters can also account for ≥2% of the output variation in each treatment scenario, as indicated by the percentages on the pie charts. These significant tendencies can also be discerned from the PRCC bar charts (the *upper panel* of Fig. 4a).

Global sensitivity analysis on the cellular ODE system also revealed important patterns (Fig. 4b). The PRCC, main and total effect indexes concurred that all the treatment scenarios were sensitive to the BMSC/preosteoblast differentiation rate (parameters *D*_MSC_ and *D*_OBp_ in Supplementary Fig. S7). In addition, I1, I1B1, and I1B4 groups were well represented by the promotion rate of IGF-1 on BMSC non-osteoblastic transformation (*i*_3_), whereas B1 and B1I4 were more sensitive to the promotion rate of BMP-2 on BMSC differentiation (*b*_1_). Interestingly, although the effective time of IGF-1 (*t*_p_) played a non-ignorable role in shaping the model output of I1, I1B1, and I1B4, its influence on B1I4 was limited, implying that the timing of cytokine delivery had much more significant influence on cell lineage progression than the effective time of a cytokine.

### Experimental validation at the molecular and cellular level

**Fig. 5:**
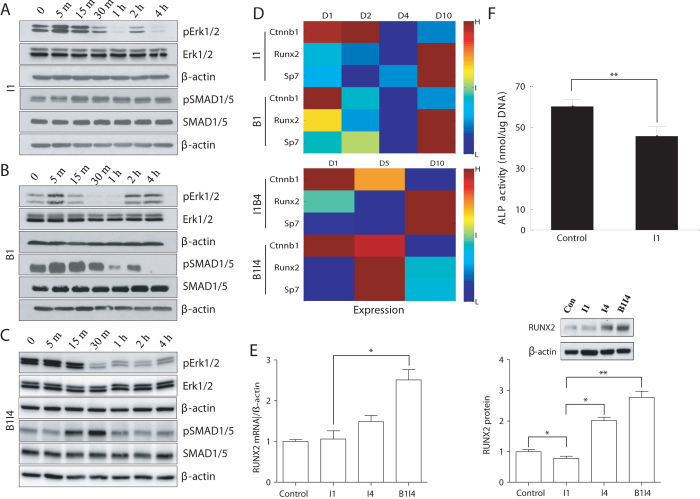
Experimental validation of critical molecular interactions and cytokine functions in W-20-17 cells. **a**–**c** Protein levels of ERK1/2, *p*ERK1/2 and SMAD1/5 and *p*SMAD1/5 determined by western blot under I1, B1 and B1I4 conditions, respectively. Proteins were harvested at day 1 for I1 and B1, and at day 4 for B1I4, immediately after the corresponding cytokine treatments. **d** Gene expression of Runx2, Sp7 (encoding osterix), and CTNNB1 (encoding β-catenin) detected at day 1, 2, 4, 10 for I1/B1 groups and at day 1, 5, 10 for I1B4/B1I4 groups from microarray data. **e** Messenger RNA and protein level of Runx2 measured by qPCR and western blot, respectively, at day 5 under control, I1, I4 and B1I4 conditions. **f** ALP activity at day 6 for I1 group and non-treated control group. Data are presented as mean ± SD (*n* = 3), *P<0.05, **P<0.001 with two-tailed Student’s *t*-test. Protein levels of *p*ERK and *p*SMAD1/5 in MC-3T3 cells treated with BMP-2/IGF-1 are shown in Supplementary Figure S11

Our investigation indicated that the most essential crosstalk between the two signaling pathways triggered by BMP-2 and IGF-1 was the interaction between ERK and SMAD. BMP-2 treatment led to the activation of SMAD1/5, while IGF-1 inhibited it through the activation of ERK1/2. We validated these predicted results with western blot. We found that the activation of ERK1/2 appeared to have an inhibiting effect on SMAD1/5 phosphorylation in I1, B1 and B1I4 treatment groups (Fig. 5a–c). This tendency was most significant in the B1I4 group (Fig. 5c), in which ERK1/2 was markedly activated and lasted for 15 min following the IGF-1 delivery, and after that, increased phosphorylation of SMAD1/5 was observed for a much longer period relative to the scenarios of single cytokine delivery as shown in Fig. 5a, b. It is worth noting that the negative regulation of ERK1/2 on SMAD1/5 appeared not strict but in an overall sense, probably because SMAD1/5 was also mediated by p38 MAPK.

The dynamic expression of relevant TFs following cytokine delivery represents another important validation of our model. We extracted the probes referring to Runx2, osterix (encoded by Sp7 gene) and β-catenin (encoded by CTNNB1 gene) from our microarray data and averaged them gene by gene (Fig. 5d). The gene expression level of β-catenin was invariably increased following IGF-1 delivery, with the B1I4 group lasting for the longest time (up to 5 days), compared to other treatment scenarios. The expression of Runx2 and osterix did not reach peak until day 10 in the I1, B1 and I1B4 groups. On the contrary, a maximum increase in the Runx2 and osterix mRNA levels was observed as early as day 5 in B1I4 group, indicating a prompt and substantial promotion of BMSC differentiation toward mature osteoblast. Our additional experiments of qPCR and western blot further confirmed that IGF-1 suppressed the osteoblastic differentiation by inhibiting expression of the osteogenic factor Runx2 (Fig. 5e). Interestingly, IGF-1 was shown to inhibit cell differentiation compared to the control, when it was delivered alone (Fig. 5f).

### Potential molecular mechanisms underlying the cytokine combination treatment

**Fig. 6:**
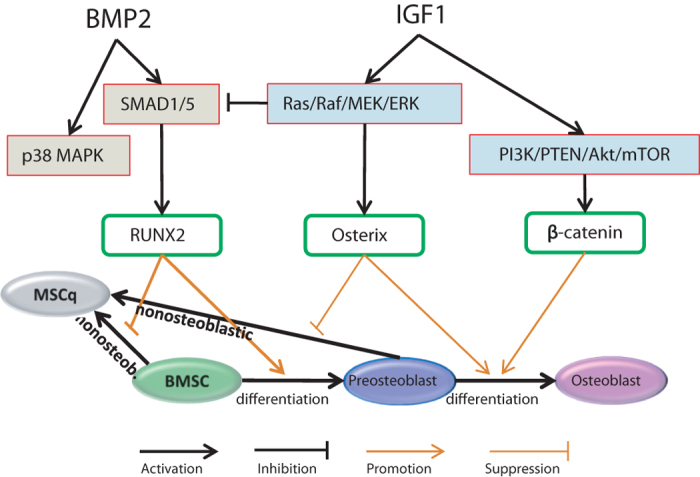
Schematic illustration of osteoblastic differentiation-related molecular mechanism under combined cytokine treatment. BMP-2 activates Runx2 through p38 MAPK and SMAD1/5 signaling. IGF-1 triggers two interactive pathways: one is the Ras/Raf/MEK/ERK pathway, which inhibits Runx2 by inhibiting SMAD1/5; the other is the PI3K/PTEN/Akt/mTOR pathway, which activates osterix and β-catenin. Runx2 is essential for early stage of BMSC differentiation, while osterix and β-catenin plays a vital role in proliferation and later stage of bone cell differentiation

Our systems biology study suggested that when IGF-1 was delivered prior to BMP-2 (i.e., before BMP-2 induced the differentiation of MSCs to preosteoblasts), IGF-1 blocked this differentiation through inhibiting SMAD1/5 and its downstream Runx2, and directed BMSCs to a non-osteoblastic lineage; and the non-osteoblastic BMSCs would never progress to bone cell lineage. Once BMSCs differentiated to preosteoblasts (directed by BMP-2), however, IGF-1 became essential in ensuring the nascent preosteoblasts further matured to osteoblasts instead of non-osteoblastic transformation. This was achieved via upregulating two critical TFs, osterix and β-catenin. In addition, IGF-1 also promoted cell proliferation by regulating cell cycle-associated molecules including ERK and p70S6K, which contributed to the sustaining bone cell growth at later stage. We illustrate this mechanism by a schematic flowchart (Fig. 6).

## Discussion

Treatment with combined cytokines, such as BMP-2 and IGF-1, represents an attractive therapeutic strategy in bone as well as other tissues regeneration.^1, 2, 24^ Although the biomaterials and bio-systems facilitating controllable cytokine delivery are becoming a routine, screening for the best cytokine (temporal) combination from numerous possibilities still poses an intensive experimental task. To address this challenge, we developed a systems biology approach (including wet-lab experiments and computational modeling) to explore the role of combined BMP-2 and IGF-1 treatment on MSC lineage progression. Based on our molecular and cellular experiments data, we constructed a multiscale model to simulate bone cell lineage progression, integrating intracellular signaling profiles and resultant transcription factor dynamic changes following treatment.

Generally, our model well reproduced the experimental observations, at both molecular and cellular levels. We indeed observed certain discrepancy between model and real RPPA data, i.e., the phosphorylation profile for the ERK protein, which exemplified very irregular pattern across the time points of measurement (especially in treatment groups I1 and B1, Supplementary Figs. S3 and S4) and could not be captured by the ODE model. Since RPPA is high-throughput data, we conducted western blot assays for ERK and SMAD and validated these significant predictions by our model (Fig. 5). Importantly, our model accurately predicted the optimal treatment scenario B1I4 (BMP-2 at day 1 followed by IGF-1 at day 4) for promising osteoblast formation, and figured out the potential molecular mechanisms underlying these experimental observations, which were largely supported by previous studies with same cell lines and growth factors.^9, 10, 22^

One of the most significant findings from our investigation is that, although IGF-1 seems to inhibit osteoblastic differentiation when delivered alone or prior to BMP-2, it enables optimal osteoblast formation when delivered at a proper timing following BMP-2 treatment. Our model suggests that IGF-1 takes effect via synergistically interacting with BMP-2 to regulate three critical bone cell-specific TFs, RUNX2, osterix and β-catenin. As elucidated above, IGF-1 and BMP-2 competitively control the expression of RUNX2, which plays an indispensable role in directing MSC to preosteoblast. Although some interactions between the involved signaling proteins have been described previously, including the inhibition of SMAD by ERK^25^ (*see* supplementary section ‘Molecular ODE system’ for more details), they were studied in separate contexts, which was not a complete mechanistic explanation for the cell lineage profiles triggered by particular cytokine combinations.

Multiscale modeling is a promising strategy for exploring system behavior regulated by components of various (physical and/or temporal) scales.^26^ In the past years, multiscale model has been widely and successfully applied in studying complex biological processes such as tumor growth and tissue regeneration.^27–32^ In our present study, the multiscale refers to multi-temporal and multi-biological scales simultaneously (Fig. 1). The biological scale includes molecular dynamic change with regard to signaling transduction and transcription factor expression, and cellular lineage progression. The temporal scale specifies time-dependent changes from minutes/hours (signaling pathway), days (transcription factor expression) to a month (cellular lineage commitment). However, in the current study, we did not consider the spatial information and hence didn’t incorporate any spatial scales or mechanical stress issues as addressed in 3D bone regeneration model.^33^ Therefore, we focused on an important aspect (the molecular mechanisms of osteoblastic differentiation and lineage) of bone regeneration, rather the whole process of bone regeneration.

The challenge of multiscale modeling is how to integrate multiple scales to represent a consolidated and well-functioning system. We addressed this issue by treating the TFs as a connecting link between the molecular signaling pathways and the cellular lineage commitment (Fig.1 and Supplementary Fig. S7). In the molecular component, growth factors BMP-2 and IGF-1 sequentially trigger the activation of different signaling pathways, further mediating the expression of bone cell-specific TFs. On the other hand, these TFs regulate various cell processes including differentiation and proliferation, and serve as perturbations of the cellular component. Therefore, the TFs play a transitional role in linking the molecular and cellular components. Since the expression of TFs changed much more slowly than the protein phosphorylation status (days vs*.* minutes), we checked the dynamic change of the immediate upstream proteins of the TFs after running the molecular system. The dynamic change of the upstream proteins represents an indicator of the presence of particular growth factors and associated TFs, which is integrated into the cellular model to account for the impact of growth factors on cell fate decision. To address the multiplicity of temporal scales in signaling transduction and resultant TFs dynamics, we treated the output of the molecular system as an initial activating status of particular TFs, and introduce a parameter to represent the effective time interval of particular cytokines and corresponding TFs (*t*_p_ in Supplementary Fig. S7).

Our model revealed a substantial heterogeneity in the signaling pathways triggered by different cytokine combinations. This was characterized by the parameter values estimated from our experimental data under individual treatments (Supplementary Fig. S2). The parameter values distributed more evenly for the treatment groups with a single cytokine (I1 and B1) in comparison with dual cytokines (I1B4 and B1I4). The extremely large or small parameters in dual delivery implied a substantially activated or inhibited signal transduction. This heterogeneous signaling transduction also reflected a dynamic change in cell composition over cell culture time. Specifically, since the RPPA values from groups I1 and B1 were obtained at day 1, while those from I1B4 and B1I4 were measured at day 4, the cellular composition was expected to have changed significantly over time. Although we could measure the preosteoblast proportion by ALP activity at early stage, it was difficult to measure all the components of a cell mixture in a real-time manner. It would be beneficial for our cell lineage model if we could define the cell-type specificity based on the microarray data. There are currently some attempts on this issue,^34^ and it will be considered in our future work when more real-time data become available.

We also conducted a systematic global sensitivity analysis on the parameters involved in our multiscale model. This strategy appeared to be more efficient in identifying the essential factors that contribute to the model output. As shown in the results (Fig. 4), the most sensitive parameters for different treatment scenarios appeared quite different, and this difference occurred in both molecular and cellular ODE systems, indicating distinct underlying mechanisms shaping the model and experiment outcomes. Particularly, despite the timing for IGF-1 treatment was critical for determining the bone lineage process, the model output turned out to be insensitive to the effective time interval (parameter *t*_p_), especially for the B1I4 scenario.

To conclude, we for the first time revealed the molecular mechanisms underlying the temporal sequence of BMP-2 and IGF-1 treatment on BMSCs, using a novel systems biology approach. Our approach proved to be promising for exploring the molecular mechanisms of multiple cytokines treatment, and consequently for identifying the optimal cytokine combination therapeutic strategy. It integrates experimental data (of different temporal/biological scales) and mathematical modeling to simulate the molecular and cellular dynamics under various treatment conditions. This strategy is powerful since it avoids intensive experimental endeavors for all possible treatment conditions to identify the optimal one. Instead, the mathematical model deals with the conditions not covered by wet-lab investigations by conducting in silico experiments. The success also depends on the deep mining of information from the genomic/proteomic and cellular data in hand, together with sufficient integration of well-established knowledge of molecular and cellular biology. We expect our approach can be parallel applied to other related research involving multiple-cytokine treatment, especially for tissue regeneration.

## Materials and methods

### Experimental studies at molecular and cellular levels

An established murine bone marrow stromal cell line, W-20-17, was obtained from the American Type Culture Collection (Rockville, MD, USA). The cells were routinely maintained in Dulbecco’s modified Eagle’s medium containing 10% bovine calf serum, 2 mM L-glutamine, 100 IU/ml penicillin, 100 μg/ml streptomycin, and 1% sodium pyruvate (all from Invitrogen, Gaithersburg, MD, USA) at 37 °C with 5% CO_2_ in air. For each treatment, 50 ng/ml IGF-1 and/or 50 ng/ml BMP-2 were added into the culture medium individually, or in combination at designated time points of each treatment scenario (Table S1).

The osteoblast-like MC3T3-E1 cells (American Type Culture Collection, Rockville, MD, USA) were cultured in the α-Minimum Essential medium (Life Technologies, Carlsbad, CA, USA) containing 10% fetal bovine serum, 100 IU/ml penicillin, and 100 μg/ml streptomycin at 37 °C under 5% CO_2_. The MC3T3-E1 cells were treated with BMP-2 and/or IGF-1 in a similar manner to the W-20-17 cells (Tables S1).

At the cellular level, we quantified the dsDNA for estimation of cell numbers, conducted alkaline phosphate (ALP) assay for measurement of osteogenic differentiation profiles, and used alizarin red S (ARS) staining to determine the extent of mineralization levels at designated time points. At the molecular level, we performed both RPPA and western blot to determine the protein level and microarray assay to check the gene expression change upon various cytokine treatments. The details of these experimental methods are provided in the Supplementary Information.

### Construction and implementation of the molecular-scale model

We employed an ODE system to describe dynamic interactions between molecular components of the inferred signaling pathways (Supplementary Fig S1A). We constructed the ODEs according to the law of mass action, which was originally proposed to explain and predict behaviors of solutions in chemical equilibrium, and proved useful in describing biochemical reactions and signaling molecules in a pathway.^35^ In our model, the signaling transduction started from binding of growth factors with their receptors to form a ligand/receptor complex, and signal to the downstream nodes through either phosphorylation or dephosphorylation of related proteins, and then regulate expression of related TFs. Thus the input and output of the system were the added growth factors and relevant TFs, respectively. Since this process typically progresses very fast (within a few hours), we assumed that the total quantity of a protein of pairing statuses (original and phosphorylated) remained constant. Likewise, the quantity of receptors (including those in the complexes) kept unchanged during the short period. This scheme substantially reduced the numbers of ODEs and parameters without any information loss. Eventually, we derived 20 ODEs for 20 signaling proteins, and additional 16 algebraic equations for extra variables corresponding to pairing statuses of corresponding proteins. The whole molecular ODE system involved only 37 parameters for the 36 variables. A detailed description of the whole molecular ODE system and involved parameters is presented in the supplementary material. Here we outline the philosophy for constructing these ODEs.

Suppose molecular *M* is activated (e.g., phosphorylated) by a series of proteins ${\{p{A}_{j}\}}_{j=1}^{m}$ and, possibly, inhibited by another batch of proteins${\{{I}_{j}\}}_{j=1}^{n}$, then the dynamic concentration [p*M*] of phosphorylated *M* can be mathematically represented by the following equation:^36^

1 $$\frac{d[pM]}{dt}=\sum _{j=1}^{m}{a}_{j}[p{A}_{j}][M]-\sum _{j=1}^{n}{d}_{j}[{I}_{j}][pM]$$

where *a*_*j*_ and *d*_*j*_ refer to phosphorylation and dephosphorylation rates exerted by p*A*_*j*_ and *I*_*j*_ respectively. And according to our assumption, the concentration of the un-phosphorylated protein *M* can be calculated by subtracting [p*M*] from the total concentration *C*_M_ of the protein, which keeps constant during the phosphorylation process, i.e., [*M*]=*C*_M_ – [p*M*]. It should be noted that the activation can alternatively be achieved through combining and forming a complex, which is depicted by differential equations analogous to (1), the only difference is the coefficients stand for association and dissociation rates. Parameters in the molecular ODE system were estimated by the RPPA data using Monte Carlo Markov Chain method,^37^ *see* Supplementary Information for detail.

### Construction and implementation of the cellular-scale model

We applied the compartmental model to simulate dynamics of cell populations. This is rational since the initial BMSCs can differentiate into a cell lineage following induction with cytokines. Hence, different cell types could concomitantly appear in the same culture system. Here the compartments include four cell types: BMSC, preosteoblast, (mature) osteoblast and non-osteoblastic cell. Upon proper stimulation, the BMSCs can differentiate to preosteoblast, which further mature to osteoblast.^38^ On the other hand, our experimental observations prompted us to hypothesize that both BMSCs and preosteoblasts may enter a non-osteoblastic state, and quit the osteoblastic lineage process if critical TFs could not be timely activated. We enhanced the benchmark compartmental model by introducing dynamic coefficients to account for the time-dependent expression levels of related TFs. In addition, the total population of all compartments does not keep constant as assumed in general, since in our study the cells are proliferating under growth factor stimulation. The compartmental model can be mathematically represented as the following ODE system (Supplementary Fig. S7):

2 $$\left\{\begin{array}{lcl} \displaystyle\frac{d[BMSC]}{dt}={P}_{MSC}(t)[BMSC]-({D}_{MSC}(t)+{Q}_{MSC}(t))[BMSC]\kern3.7pc\\ \displaystyle\frac{d[MSCq]}{dt}={Q}_{MSC}(t)[BMSC]+{Q}_{OBp}(t)[OBp]\kern8.5pc\\ \displaystyle\frac{d[OBp]}{dt}={P}_{OBp}(t)[OBp]+{D}_{MSC}(t)[BMSC]-({D}_{OBp}(t)+{Q}_{OBp}(t))[OBp]\\ \displaystyle\frac{d[OBa]}{dt}={P}_{OBa}(t)[OBa]+{D}_{OBp}(t)[OBp]\kern10pc\end{array}\right.$$

In Eq. (2), “BMSC”, “OBp”, “OBa” and “MSCq” stand for mouse bone marrow-derived mesenchymal stem cell, preosteoblast, osteoblast and non-osteoblastic cell respectively; the proliferation rate P_*_(*t*), differentiation rate D_*_(*t*), and non-osteoblastic transformation rate Q_*_(*t*) for different cell types are time-dependent, and represented by a basic rate plus joint effects (promotion or inhibition, or both) exerted by related growth factors. Parameters in the cellular ODE system were estimated by the ARS (for OBa) and dsDNA (for total cell mass) data using regular least square optimization, *see* details in the Supplementary Materials.

### Availability of data and materials

Original experimental data and supplementary materials/methods can be found online as Supplementary Information. Matlab codes are available upon request.

## Electronic supplementary material

Supplementary Information

Supplementary Dataset 1

Supplementary Dataset 2

Supplementary Dataset 3
