# Supplementary material for: A systems biology approach to studying the molecular mechanisms of osteoblastic differentiation under cytokine combination treatment
Source: NPJ Regen Med. 2017 Mar 10;2:5. doi: 10.1038/s41536-017-0009-0 (PMC5677954; doi:10.1038/s41536-017-0009-0)
Supplement: Supplementary file 1 — Supplementary Information [file 41536_2017_9_MOESM1_ESM.pdf]

Supplementary Information for

A systems biology approach to studying the molecular mechanisms of osteoblastic differentiation under cytokine combination treatment

Hua Tan[1, †], Ruoying Chen[1, †], Wenyang Li[1,2, †], Weiling Zhao[1], Yuanyuan Zhang[3], Yunzhi Yang[4,5,6,*], Jing Su[1], and Xiaobo Zhou[1,*]

[1]Center for Bioinformatics & Systems Biology, Department of Radiology, Wake Forest University School of Medicine, Winston-Salem, NC 27157, USA

[2]Chongqing Key Laboratory of Oral Diseases and Biomedical Sciences and College of Stomatology, Chongqing Medical University, Chongqing 400016, China

[3]Institute of Regenerative Medicine, Wake Forest University School of Medicine, Winston-Salem, NC 27157, USA

[4]Department of Orthopedic Surgery, Stanford University, Stanford, CA 94305, USA

[5]Department of Materials Science and Engineering, Stanford University, Stanford, CA 94305, USA

[6]Department of Bioengineering, Stanford University, Stanford, CA 94305, USA

[†]Co-first authors

Summary of supporting information

This file includes:

Supplementary experimental methods

Supplementary computational methods

Supplemental Tables

Supplemental Figures

Supplementary references

Other materials as separate files:

Dataset S1. A summary of the DEGs, DAPs and UTRs obtained from the microarray, RPPA and IPA analysis respectively. The DEGs, DAPs and UTRs were pooled together and duplicates were removed. These molecules were uploaded into the IPA system for canonical pathways mapping analysis. DEGs: differentially expressed genes; DAPs: differentially activated proteins; UTRs: upstream regulators.

Dataset S2. Raw data for reverse phase protein array (RPPA). In sample description, D1 means samples measured on day 1; I1_B4 means IGF-1 was added at day 1, and BMP-2 was added at day 4; the ending numbers indicate protein levels were measured at 0 (control), 5, 15, 30, 60, 120, 240 minutes following treatment. For antibody description, Akt_pS473-R-V_GBL9016996 means this antibody specifically recognizes phosphorylated Akt on Serine 473, and this is a rabbit (-R) antibody validated (-V) for RPPA application. GBL9016996 is the slide ID (barcode).

Dataset S3. Raw data for microarray. Each row refers to one probe, and each column corresponds to an experimental condition. For instance, 'D1.IGFControl.BMPControl' is the control group measured at day 1, while 'D5.IGFD4.BMPD1' stands for 'B1I4' measured at day 5.

Supplementary experimental methods

Double strand DNA (dsDNA) quantitation

Double strand DNA (dsDNA) was used for estimation of cell numbers using Quant-iT PicGreen assay kit (Invitrogen). Briefly, 30,000 cells/well were plated into 24-well plates at a density of 30,000 cells per well and allowed to attach for 24 h. As we previously did, IGF-1 (50 ng/ml) and/or BMP-2 (50 ng/ml) were added into the culture medium on day 1, 2, 4, and 6. At day 1, 4, 8, 11, and 29, culture medium was removed and cells washed three times with 1 X PBS. Five hundred μl of 0.05% Triton-X in PBS was used to lyse cells. Cell lysates were stored at −80°C until further analysis. Each thawed sample was sonicated for 5 s using an ultrasonic cell disrupter (Fisher Scientific, pittsburgh, PA). Fifty μl of the cell lysates were mixed with an equal volume of PicoGreen working solution and incubated for 5 min in the dark. The plate was then read on SpectraMax M2 fluorescence microplate reader (Molecular Devices Inc., Sunnyvale, CA) at excitation and emission wavelengths of 485 and 535 nm, respectively. The dsDNA contents were calculated according to a standard curve generated using double stranded DNA standard.

Alkaline phosphatase (ALP) assay

ALP is an early osteogenic differentiation marker and its activity was determined using p-nitiophenyl phosphate (p-NPP) substrate in a colorimetric assay. Briefly, 50 μl aliquots of the cell lysates were placed in a 96-well plate and an equal volume of working reagent was added to each well. The working reagent consisted of 0.5 M 2-amino-2-methyl-1-proanol (Sigma), 6.6 mM p-nitrophenyl phosphate (Sigma), and 0.33mM magnesium chloride. The mixtures were then incubated for 1~3 h at 37℃. After incubation, 100μl of 1M sodium hydroxide was used to stop the reaction. ALP activity was determined from the absorbance of a standard curve prepared using p-nitrophenol standard (Sigma). The absorbance was measured at 405 nm using the SpectraMax M2 microplate reader. The ALP activity was determined by normalizing ALP value of each sample to its dsDNA content.

Alizarin Red S (ARS) staining

To determine the extent of mineralization, the cultured cells were stained with Alizarin Red Staining solution on day 28. Briefly, the culture medium was removed from each well and washed twice with 1 X PBS. The cells were then fixed with 10% formaldehyde, for 15 min at room temperature and rinsed three times with distilled water. Two hundred μl of Alizarin Red stain Solution (Sigma-Aldrich, St. Louis, MO) was added into each well and the cells were incubated at room temperature for 20 min. The cells were destained with 400μl 10% acetic acid for 30 min at room temperature and then transferred into 1.5 mL microcentrifuge tubes. After vortexing, the slurry was overlaid with 500 μl of mineral oil (Sigma–Aldrich), heated at 85℃ for 10 min, and cooled down on ice for 5min. Four hundred μl of the supernatant was transferred to a new 1.5mL microcentrifuge tube and neutralized with 150μl of 10% Ammonium hydroxide to the range of pH 4.1-4.5. One hundred and fifty μl of standard/sample solution was added to an opaque-walled, transparent bottom 96-well plate. The absorbance of samples was measured at a 405nm wavelength using the SpectraMax M2 microplate reader.

Reverse phase protein array (RPPA)

Cells were harvested at 5', 15', 30', 1h, 2h, and 4h after cytokine treatments with 4 replicates. After washing twice with 1 X PBS, cells were lysed with RIPA buffer (150 mM NaCl, 1% NP-40, 1% sodium deoxycolate in 50 mM Tris-HCl, pH 7.5). Then cells were scraped and collected into microcentrifuge tubes. The cell lysates were then centrifuged at 14,000 rpm for 10 min at 4℃, and supernatants were collected and protein concentration was determined by Bradford method (Biorad, Hercules, CA). The cell lysates were mixed with SDS sample buffer without bromophenol blue and boiled for 5 min. The samples were then sent to RPPA core facility in MD Anderson Cancer Center for analysis.

RNA extraction and microarray assay

Total RNA was isolated at day 1, 2, 4, 5 and 10 after cytokine treatment using RNeasy mini kit according to the protocol supplied by the manufacturer (Qiagen, Carlsbad, CA); the concentration of RNA was determined spectrophotometrically at 260 nm. The total RNA was reverse-transcribed and amplified with biotin-labeled using Illumina Total Prep RNA Amplification Kit (Ambion, Austin, TX), and hybridized with mouse WG-6 v2.0 Expression BeadChip (Illumina, San Diego, CA).

Western blot assay

Cells were lysed using RIPA buffer containing proteinase and phosphatase inhibitors (see RPPA section). Twenty μg of cell lysates was mixed with an equal volume of sample buffer, denatured by boiling, and then separated on a 10–15% polyacrylamide mini-gel. The proteins were transferred onto a PVDF membrane (Bio-Rad Laboratories). After blocking with 5% milk for 1h, the blots were incubated overnight with ERK, pERK, SMAD1/5, pSMAD1/5 and β-actin antibodies (all of the antibodies were purchased from Cell Signaling Technology, Danvers, MA, USA). The blots were then incubated with anti-mouse or anti-rabbit IgG horseradish peroxidase-conjugated antibodies (Cell signaling) for 1h at room temperature. Finally, the signal was detected using ECL Plus reagents (Pierce, Rockford, IL).

Supplementary computational methods

Signaling pathway inference

To obtain a generic signaling pathway related to BMSC differentiation and proliferation, it is the initial step to identify the relevant molecular components that potentially take part in the signaling transduction under investigation. We achieved this based on the mRNA microarray data and reverse phase protein array (RPPA) data. First, we extracted the differentially expressed genes (DEGs) under each treatment condition by setting an intensity variation threshold $\theta g=1$ after $\log_2$ transformation of the original microarray data. Likewise, we collected the differentially activated proteins (DAPs) from the RPPA data, with a fold change threshold $\theta p=1.5$. This procedure yielded a batch of molecules (genes/proteins) that were potentially associated with responses of the cells to various cytokine treatments. These molecules were then uploaded to IPA (http://www.ingenuity.com) for upstream analysis. A collection of upstream regulators (UTRs) was obtained from IPA analysis. All of the obtained DEGs, DAPs and their UTRs (Dataset S1) were then mapped to the canonical cell signaling pathways of IPA. The final generic pathway was determined by integrating the pathways that were well overlapped with IPA canonical pathways, especially the BMP and IGF-1-initiated signaling cascades (supplementary materials). In this

procedure, we also took into account the existing literatures to refine the cross-talked signaling pathway (Fig. S1A) for further simulation and analysis.

Molecular ODE system

Twenty proteins and 36 variables were included in the signaling pathway equations. Here the phosphorylation status of a protein was treated as an independent variable. Our analysis indicated that BMP-2 and IGF-1 trigger the activation of different signaling pathways and converge at some nodes, exerting a joint effect on the bone cell lineage process. We obtained the generic signaling pathway based on our molecular data, with critical nodes modified and/or confirmed by retrieving existing literatures. To be specific, BMP-2 binds to its receptor (denoted 'BMP2R'), and activates TAK1-p38 MAPK and SMAD1/5 signaling pathways simultaneously, and eventually converge at the transcription factor Runx2 [1-3]. Likewise, IGF-1 binds to its receptor (denoted 'IGF1R') and starts a series of ensuing events, including the concurrent activation of the Ras/Raf/MEK/ERK and PI3K/PTEN/Akt/mTOR cascades. These two cascades coordinate to play important role in bone cell proliferation, apoptosis and differentiation [4, 5]. Their effects on bone cell lineage commitment are achieved by regulating essential transcription factors such as Runx2, osterix and β-catenin. IGF-1 can inhibit Runx2 via activating ERK, which is a significant crosstalk between BMP-2 and IGF-1 triggered signaling pathways [6]. Osterix was widely considered as downstream of Runx2, but some research implied that it also might be mediated by ERK [7]. Furthermore, the IGF-1 signaling involves stabilization of β-catenin through inhibiting GSK-3-mediated β-catenin phosphorylation [5, 8], and regulation of cell cycle related transcription factors via ERK and ribosomal protein S6 stimulation [9, 10].

We employed the following system of ordinary differential equations (ODEs) and algebraic equations (1) to describe the dynamic change of molecules involved in the bone cell specific signaling pathways. The input for this ODE system was the dosage of BMP-2 and/or IGF-1 reflecting the growth factor combinations; while the output is the dynamic change of all participating proteins. The total concentration of each protein (including dual statuses: phosphorylated and un-phosphorylated) was assumed to keep unchanged during such a short period – the signaling proteins were typically activated within a few minutes and lasted for up to several hours. In this way, we dramatically reduced the number of ODEs and parameters without any information loss. Here, [protein] refers to the concentration of the understudied protein, while the symbols of the proteins (variables) and the association/dissociation rates (parameters) are illustrated in Fig. S1A. The equations are derived based on various molecular interactions as illustrated in Fig. S1B, as we previously did [11, 12].

$$\frac{d[BMP2]}{dt} = -a_1[BMP2][BMP2R] + d_1[BMP2RC] \qquad \text{(S1)}$$

$$\frac{d[BMP2RC]}{dt} = a_1[BMP2][BMP2R] - d_1[BMP2RC],\ [BMP2R] = C_{BMP2R} - [BMPR2C]$$

$$\frac{d[\mathrm{pTAK1}]}{dt} = a_2[\mathrm{TAK1}](1+[BMP2RC]) - d_2[\mathrm{pTAK1}],\ [\mathrm{TAK1}] = C_{TAK1} - [\mathrm{pTAK1}]$$

$$\frac{d[\mathrm{pp38}]}{dt} = a_3[\mathrm{p38}][\mathrm{pTAK1}] - d_3[\mathrm{pp38}],\ [p38] = C_{p38} - [\mathrm{pp38}]$$

$$\frac{d[\text{pSMAD1}]}{dt} = a_4[\text{SMAD1}](1+[\text{BMP2RC}]) - d_4[\text{pSMAD1}][\text{pERK}]\ [\text{SMAD1}] = C_{SMAD1} - [\text{pSMAD1}]$$

$$\frac{d[IGF1]}{dt} = -a_6[\text{IGF1}][\text{IGF1R}] + d_6[\text{IGF1RC}]$$

$$\frac{d[IGF1RC]}{dt} = a_6[\text{IGF1}][\text{IGF1R}] - d_6[\text{IGF1RC}],\ [\text{IGF1R}] = C_{IGF1R} - [\text{IGF1RC}]$$

$$\frac{d[\text{pIRS1}]}{dt} = a_7[\text{IRS1}](1+[\text{IGF1RC}]) - d_7[\text{pIRS1}],\ [\text{IRS1}] = C_{IRS1} - [\text{pIRS1}]$$

$$\frac{d[Ras]}{dt} = a_8(2-[\text{Ras}])[\text{pIRS1}] - d_8[\text{Ras}]$$

$$\frac{d[\text{pc}-Raf]}{dt} = a_9[\text{c}-Raf][\text{Ras}] - d_9[\text{pc}-Raf],\ [\text{c}-Raf] = C_{c-Raf} - [\text{pc}-Raf]$$

$$\frac{d[\text{pMEK}]}{dt} = a_{10}[\text{MEK}][\text{pc}-\text{Raf}] - d_{10}[\text{pMEK}],\ [\text{MEK}] = C_{MEK} - [\text{pMEK}]$$

$$\frac{d[\text{pERK}]}{dt} = (a_{11}[\text{pMEK}] + x_2[\text{pAKT}])[\text{ERK}] - d_{11}[\text{pERK}],\ [\text{ERK}] = C_{ERK} - [\text{pERK}]$$

$$\frac{d[\text{p}PDK1]}{dt} = a_{12}[PDK1][\text{PI3K}] - d_{12}[\text{p}PDK1],\ [\text{PDK1}] = C_{PDK1} - [\text{p}PDK1]$$

$$\frac{d[\text{pGSK}3\beta]}{dt} = a_{13}[\text{GSK}3\beta][\text{pAKT}] - d_{13}[\text{pGSK}3\beta],\ [\text{GSK}3\beta] = C_{GSK3\beta} - [\text{pGSK}3\beta]$$

$$\frac{d[\text{pPTEN}]}{dt} = a_{14}[PTEN] - d_{14}[\text{p}PTEN],\ [\text{PTEN}] = C_{PTEN} - [\text{p}PTEN]$$

$$\frac{d[\text{PI3K}]}{dt} = a_{15}(2-[\text{PI3K}])[\text{pIRS1}] - d_{15}[\text{PI3K}]$$

$$\frac{d[\text{pAKT}]}{dt} = (a_{16d}[\text{pPDK1}] + a_{16i}[PI3K] + \text{x}_1(1+BMP2RC))[\text{AKT}] - d_{16}[\text{pAKT}][\text{pPTEN}]$$

$$[\text{AKT}] = C_{AKT} - [\text{pAKT}]$$

$$\frac{d[\text{pmTOR}]}{dt} = a_{17}[\text{mTOR}][\text{pAKT}] - d_{17}[\text{pmTOR}],\ [\text{mTOR}] = C_{mTOR} - [\text{pmTOR}]$$

$$\frac{d[\text{pp70}]}{dt} = a_{18}[\text{p70}][\text{pmTOR}] - d_{18}[\text{pp70}],\ [\text{p70}] = C_{p70} - [\text{pp70}]$$

$$\frac{d[\text{pS6}]}{dt} = a_{19}[\text{S6}][\text{pp70}] - d_{19}[\text{pS6}],\ [\text{S6}] = C_{S6} - [\text{pS6}]$$

We fitted the ODE system (S1) to the RPPA dynamic data (collected shortly after the cytokine treatment at 0, 5, 15, 30, 60, 120, 240 minutes) to estimate the parameters using the Markov Chain Monte Carlo (MCMC) method (see main text). Figure S2 illustrates a summary of the parameters estimated for each treatment separately, see also Table S2. Figures S3-S6 shows the fitting and prediction results of protein dynamics under different treatments as denoted in each panel.

Cellular ODE system

We constructed the following ODE system to depict the cellular lineage dynamics regulated by the combination of BMP-2 and IGF-1 (Fig. S7). The coefficients with regard to cell differentiation D*(t), proliferation P*(t) and non-osteoblastic rate Q*(t) were also dynamic, reflecting the real-time profiles of related transcription factors (TFs), which were indicative of the presence of associated growth factors. We figured out a formula for each coefficient according to our experimental data and the biological functions of the growth factors.

$$\begin{cases} \frac{d[\mathrm{BM}\,SC]}{dt} = P_{MSC}(\mathrm{t})[\mathrm{BMSC}] - (\mathrm{D}_{MSC}(t) + Q_{MSC}(t))[\mathrm{BMSC}] \\ \frac{d[MSCq]}{dt} = Q_{MSC}(\mathrm{t})[\mathrm{BMSC}] + Q_{OBp}(\mathrm{t})[\mathrm{OBp}] \\ \frac{d[\mathrm{OBp}]}{dt} = P_{OBp}(\mathrm{t})[OBp] + \mathrm{D}_{MSC}(\mathrm{t})[\mathrm{BMSC}] - (\mathrm{D}_{OBp}(t) + Q_{OBp}(\mathrm{t}))[\mathrm{OBp}] \\ \frac{d[\mathrm{OBa}]}{dt} = P_{OBa}(\mathrm{t})[OBa] + \mathrm{D}_{OBp}(\mathrm{t})[\mathrm{OBp}] \end{cases} \quad \text{(S2)}$$

In these equations, "BMSC" refers to initial bone marrow stromal cell, "OBp" and "OBa" stand for preosteoblast and osteoblast respectively, and "MSCq" represents non-osteoblastic cell transformed from BMSC or preosteoblast. Since cell death was not our focus in the present study (we were focusing on the cell differentiation/growth with and without cytokine stimulation), and cells were cultured for a relatively short period of time, we did not introduce an independent term and parameter for cell death. Instead, we implicated the cell death in the proliferation term, i.e., the proliferation rate will be calibrated by the experimental data to account for the potential cell death.

Dynamic proliferation rate: First, we assumed that the cell basic proliferation rate $d_r(t)$ decreases exponentially with time t and can be mathematically represented by $d_r(t) = \alpha \cdot e^{-\beta \cdot t}$. Correspondingly, the total cell mass $G(t)$ at time t could be calculated as (the initial cell mass has been normalized to unity):

$$\mathrm{G}(t) = \prod_{i=1}^{t}(1 + \mathrm{d}_r(\mathrm{i})) \quad \text{(S3)}$$

Where, Π is the product notation, and $d_r(i)$ refers to a dynamic proliferation rate over time instead of a constant one. It should be noted that this population growth model is comparable to the logistic growth equation with the difference lying in the time-dependent growth rate $d_r(\mathrm{t})$. Specifically, in the logistic model, the rate of proliferation rate (normalized to the population size) linearly decreases with population size; while in our model, the rate decreases exponentially with time. Since we are describing a population expansion process within a very limited space and environment range, the population growth vanishes in a short period of time (<1 month) due to cell confluence. We found that the

exponentially decreased rate fits the data better comparing with the linearly decreased rate. We fitted this formula to the total cell mass data (dsDNA) and obtained the coefficients α=0.1156, β=0.0969.

To get the dynamic proliferation rate enhanced by growth factor IGF-1, we introduced a parameter i_1 to account for the promotion effect of IGF-1 on cell proliferation and adjusted the basic proliferation to the following formulae:

$$\begin{cases} P_{MSC}(t) = (1+\frac{\mathrm{i}_1}{10}\cdot \chi_{\Delta t<t_p})\cdot \mathrm{d}_r(t) \\ P_{OBp}(t) = (1+\frac{\mathrm{i}_1}{5}\cdot \chi_{\Delta t<t_p})\cdot \mathrm{d}_r(t) \\ P_{OBa}(t) = (1+\mathrm{i}_1\cdot \chi_{\Delta t<t_p})\cdot \mathrm{d}_r(t) \\ P_{MSCq}(t) = \mathrm{d}_r(t) \end{cases} \tag{S4}$$

Here t_p is effective time of IGF-1 (i.e., the effect of IGF-1 on cells is assumed to last for a maximum time period of t_p), and $\chi_{\Delta t<t_p}$ is a characteristic function indicating whether the IGF-1 is in effective time period, represented by the following formula:

$$\chi_{\Delta t<t_d} = \begin{cases} 1, \Delta t < t_d \ (\Delta t = \textit{time duration following cytokine delivery}) \\ 0, \textit{otherwise} \end{cases} \tag{S5}$$

The constants multiplied to i_1 stand for different promotion efficiency of IGF-1 on particular cell types. We assumed IGF-1 cannot promote proliferation of the non-osteoblastic cells ('MSCq') and left it as the basic proliferation rate.

Dynamic differentiation/non-osteoblastic rate: For BMSC and preosteoblast, there are another two cell fates at each time point besides proliferation: differentiation or non-osteoblastic switch. We set basic proportion for each of the two cell fates at initial time, i.e., if the initial differentiation rate for BMSC and preosteoblast is D_{MSC} and D_{OBp}, then the corresponding non-osteoblastic transformation rate is (1-D_{MSC}) and (1-D_{OBp}). Denote the promotion rate of BMP-2 on BMSC differentiation as b_1 and inhibition rate of IGF-1 on BMSC differentiation as i_3; furthermore, let i_2 be the promotion rate of IGF-1 on preosteoblast differentiation. Then the dynamic differentiation and non-osteoblastic transformation rate for BMSC and preosteoblast can be respectively represented as

$$\begin{cases} D_{MSC}(t) = \gamma_1 \cdot d_{MSC}(t) \\ Q_{MSC}(t) = \gamma_1 \cdot (1-d_{MSC}(t)) \\ \textit{where, } d_{MSC}(t) = D_{MSC}\cdot(1+b_1\cdot\chi_{\Delta t<t_d})\cdot(1-i_3\cdot\chi_{\Delta t<t_p}) \end{cases} \tag{S6}$$

Likewise,

$$\begin{cases} D_{OBp}(t) = \gamma_2 \cdot d_{OBp}(t) \\ Q_{OBp}(t) = \gamma_2 \cdot (1-d_{OBp}(t)) \\ \textit{where, } d_{OBp}(t) = D_{OBp}\cdot(1+i_2\cdot\chi_{\Delta t<t_p}) \end{cases} \tag{S7}$$

Here γ_1, γ_2 are scaling constants accounting for percentage of cells going to differentiation and non-osteoblastic transformation status respectively at each time point (γ_1=0.062, γ_2=0.1 in our calculation, estimated from assuming that 99% of the BMSCs will be exhausted within 2 weeks due to differentiation and non-osteoblastic transformation). And $\chi_{\Delta t<t_d}$ is a characteristic function indicating whether the BMP-2 is in its effective time period, defined similar to formula (S5).

We performed a gird search of the parameters by minimizing the difference between the experimental cellular data and model prediction. The involved parameters and their values are summarized in Table S3. The experimental and model prediction results for various treatment conditions are illustrated in Fig. S8.

Parameter estimation of the multiscale model

The molecular ODE system involved 20 differential equations and 39 parameters. The 20 ODEs corresponded to 20 different proteins, 12 of which had dynamic RPPA data. We adopted the Markov chain Monte Carlo (MCMC) method [37] to determine the optimal parameters $\hat{\theta}$ that minimize the difference between real data and model prediction. The MCMC method is an efficient heuristic algorithm especially suitable for large-scale problem. We solved the following minimization problem

$$\hat{\theta} = \arg\min_{\theta\in\Theta} \sum_{i}^{N} w_i \sum_{t=1}^{T} v_t (\mathrm{y}_i(\mathrm{t},\theta) - \mathrm{y}_i^t)^2 \tag{S8}$$

Where $\mathrm{y}_i(\mathrm{t},\theta)$ and y_i^t represent the quantity of molecule *i* at time point *t* for model prediction (obtained with parameter set θ) and experimental measurement, respectively; w_i and v_t correspond to protein and time-point weights; and *N* and *T* refer to the number of proteins and time points, respectively; Θ represents the parameter space with a reasonable range.

The cellular ODE system included only 4 equations and 8 parameters. We performed a grid search from a desired range for each parameter. We set coarse grid first to obtain initial optimal parameters and then conducted a finer grid search around the initial value for each parameter. The objective function for the cellular ODEs was similar to the molecular ones, except that the *y*'s in formula (S8) represent cell numbers instead of protein concentrations.

Global sensitivity analysis on model parameters

We conducted a global sensitivity analysis on all the parameters to see how the changes on parameters will influence the output variation. It is different from the regular sensitivity analysis, which changes one parameter by certain percentage of its baseline value at one time. The global sensitivity analysis perturbs all the involved parameters simultaneously along particular sampling curves [23]. This procedure will result in an output variation which is a mixture of various frequencies. By variance decomposition of the model output, we can identify the factors (parameters) that significantly contribute to the output variation (a percentage accounted for by each parameter). To measure global sensitivity, we assumed that each parameter is perturbed by a uniformly distributed random variable within the range of ±10% of the nominal parameter value obtained through data fitting, and set the model output as

the difference between real data and model prediction. Then we computed the Spearman's partial rank correlation coefficients (PRCC) from 100 samples generated with a Latin Hypercube sampling (LHS) algorithm [38]. We also calculated the main (first order sensitivity index S_i) and total effect (total-order sensitivity index S_{Ti}) of each parameter on model output using the extended Fourier Amplitude Sensitivity Test (eFAST) method [39]. The total number of model simulations in eFAST is given by $N = N_S \times k \times N_R$, where N_S=65, N_R=5, and k is the number of parameters analyzed.

Supplementary Tables

Table S1. Summary of the temporal combinatorial design of growth factor treatments

Growth Factor	Treatment Time Point /day					
**IGF-1**	-	1	-	1	1	4
**BMP-2**	-	-	1	1	4	1
**Label**	Control	I1	B1	I1B1	I1B4	B1I4

IGF-1: insulin-like growth factor 1; BMP-2: bone morphogenetic protein 2; '-' indicates no treatment with that cytokine.

Table S2. Summary of parameters involved in the molecular ODE system

Parameters*	D1-I1	D1-B1	D4-I1B4	D4-B1I4
**a1**	0.045988	0.031338	0.011579	0.049261
**d1**	0.034274	0.025853	0.08761	0.067687
**a2**	0.062813	0.058484	0.052087	0.023775
**d2**	0.041141	0.067188	0.068641	0.048339
**a3**	0.052615	0.040207	0.042515	0.066759
**d3**	0.039464	0.035458	0.012766	0.052297
**a4**	0.060058	0.054319	0.066695	0.019845
**d4**	0.069343	0.015103	0.054334	0.02934
**a6**	0.086012	0.025477	0.054715	0.062483
**d6**	0.054339	0.049774	0.019846	0.068706
**a7**	0.037933	0.09415	0.076963	0.029383
**d7**	0.060721	0.069632	0.062804	0.01716
**a8**	0.054526	0.055543	0.080291	0.048362
**d8**	0.017553	0.080199	0.076632	0.065051
**a9**	0.041184	0.058382	0.035503	0.045538
**d9**	0.060093	0.051659	0.043331	0.052511
**a10**	0.041196	0.009017	0.080256	0.058309
**d10**	0.06236	0.058263	0.058338	0.036691
**a11**	0.049104	0.050522	0.054879	0.099729
**d11**	0.039159	0.078251	0.035257	0.102675
**a12**	0.045159	0.02302	0.065295	0.047747
**d12**	0.045902	0.027468	0.022519	0.028638

**a13**	0.042137	0.036031	0.08724	0.087877
**d13**	0.047753	0.060005	0.007341	0.002567
**a14**	0.035194	0.022602	0.049309	0.055384
**d14**	0.044633	0.041503	0.05914	0.091076
**a15**	0.037749	0.035376	0.039866	0.02467
**d15**	0.048399	0.062207	0.078601	0.046425
**a16d**	0.028954	0.032982	0.019872	0.061465
**a16i**	0.035513	0.056147	0.003224	0.008847
**d16**	0.056872	0.065995	0.044747	0.082669
**a17**	0.035718	0.030335	0.057999	0.085855
**d17**	0.054742	0.054571	0.027718	0.054986
**a18**	0.054436	0.063707	0.070328	0.088336
**d18**	0.045229	0.042829	0.072296	0.031611
**a19**	0.034544	0.03817	0.045497	0.036988
**d19**	0.083632	0.068031	0.067683	0.028236
**x1**	0.018138	0.063432	0.027347	0.019899
**x2**	0.028533	0.037762	0.017556	0.07048

*The physical meaning of the parameters are presented in Equation (S1) and Figure S1. ai and di refer to association and dissociation rate respectively for protein i, and x1 (x2) represents possible activating effect of BMP2RC (Akt) on Akt (ERK1/2). See also Materials and Methods in the main text.

Table S3. Summary of parameters involved in the cellular lineage ODE system

Parameter*	Description	Baseline	Range
D_{MSC}	Relative differentiation rate of BMSC	0.5	[0.45, 0.55]
D_{OBp}	Relative differentiation rate of preosteoblast	0.5	[0.45, 0.55]
b_1	Promotion rate of BMP-2 on BMSC differentiation	0.9	[0.81, 0.99]
i_1	Promotion rate of IGF-1 on cell proliferation	0.25	[0.225, 0.275]
i_2	Promotion rate of IGF-1 on preosteoblast differentiation	0.9	[0.81, 0.99]
i_3	Promotion rate of IGF-1 on BMSC non-osteoblastic transformation	0.8	[0.72, 0.88]
t_d	Effective time of BMP-2 (hours)	24	[22, 26]
t_p	Effective time of IGF-1 (hours)	12	[11, 13]

*These parameters apply to all the six treatment scenarios listed in table S1. The parameters listed here mainly account for the effects of growth factors on cell activities, as illustrated in Fig. S7.

Supplementary Figures

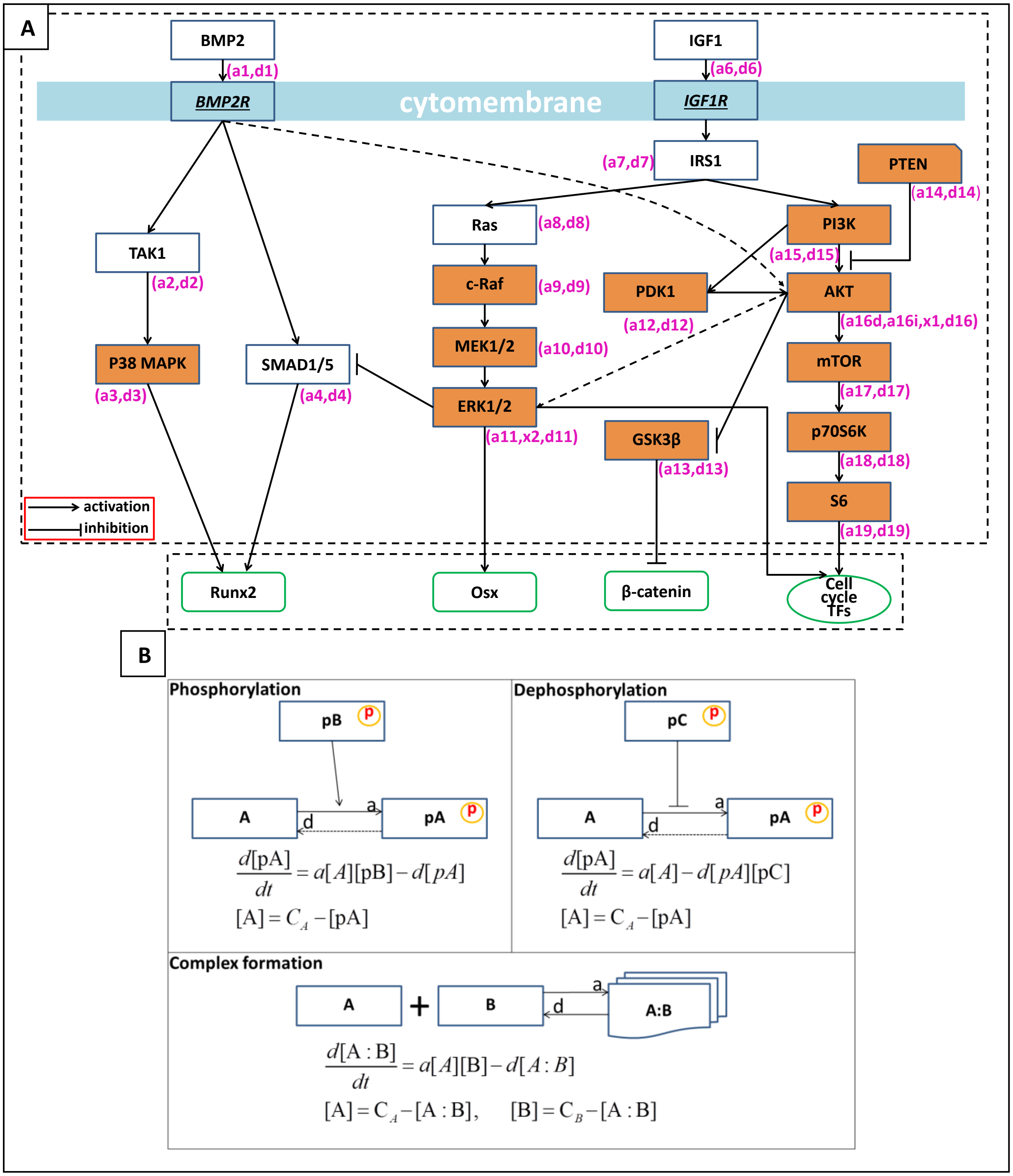

$$\frac{d[\mathrm{pA}]}{dt} = a[A][\mathrm{pB}] - d[pA]$$
$$[\mathrm{A}] = C_A - [\mathrm{pA}]$$

$$\frac{d[\mathrm{pA}]}{dt} = a[A] - d[pA][\mathrm{pC}]$$
$$[\mathrm{A}] = \mathrm{C}_A - [\mathrm{pA}]$$

$$\frac{d[\mathrm{A:B}]}{dt} = a[A][\mathrm{B}] - d[A:B]$$
$$[\mathrm{A}] = \mathrm{C}_A - [\mathrm{A:B}], \quad [\mathrm{B}] = \mathrm{C}_B - [\mathrm{A:B}]$$

Figure S1. Molecular signaling pathway (A) and the ODE construction philosophy (B). In (A), a_i and d_i refer to association and dissociation rate respectively for protein i as denoted in the boxes, and x_1 (x_2) represents possible activating effect of BMP2RC (Akt) on Akt (ERK1/2). The proteins with RPPA data available are coded by orange background.

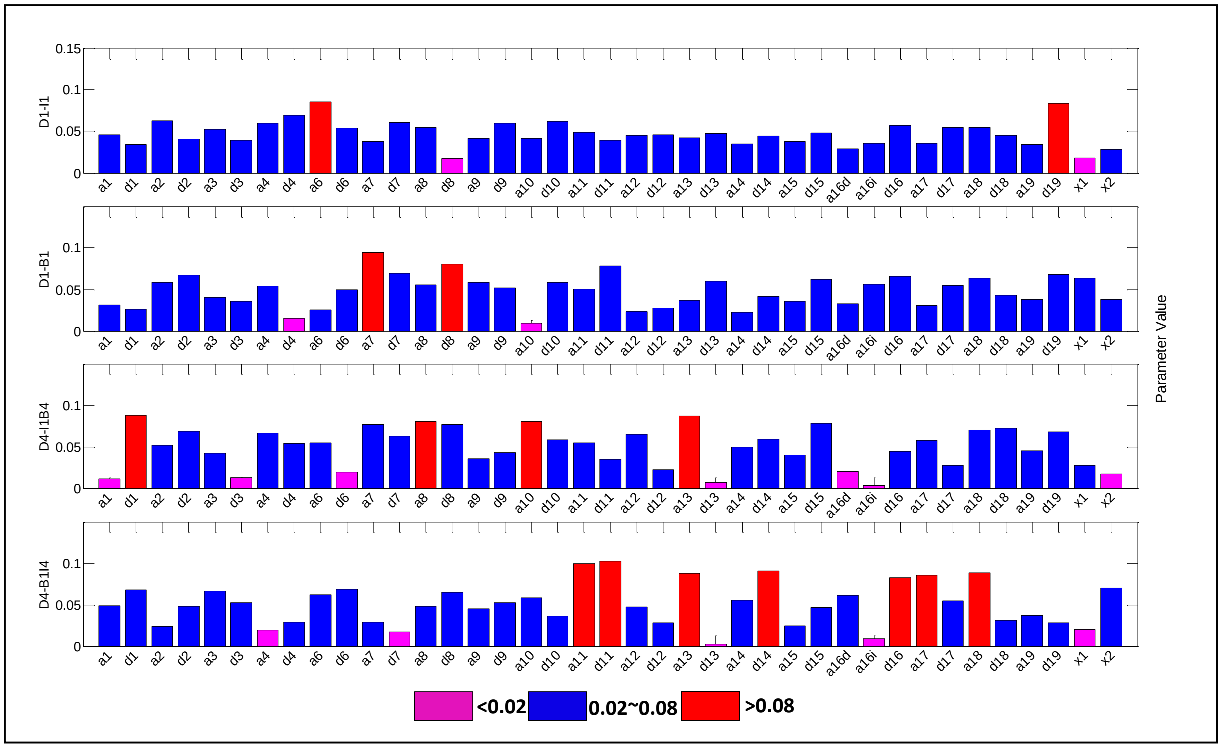

Figure S2. Illustrative summary of parameter values used in the molecular ODE system**.** These parameters are separately estimated from the RPPA data of four treatment conditions corresponding to figure S3-S6 respectively. The parameters take different values in each treatment scenario, indicating heterogeneous profiles of signaling activation/inhibition under various treatment conditions (denoted on the y-axes; D1- and D4- indicate that RPPA was measured on day 1 and day 4, respectively). Parameters falling in different ranges are encoded by particular colors, as illustrated by the legend.

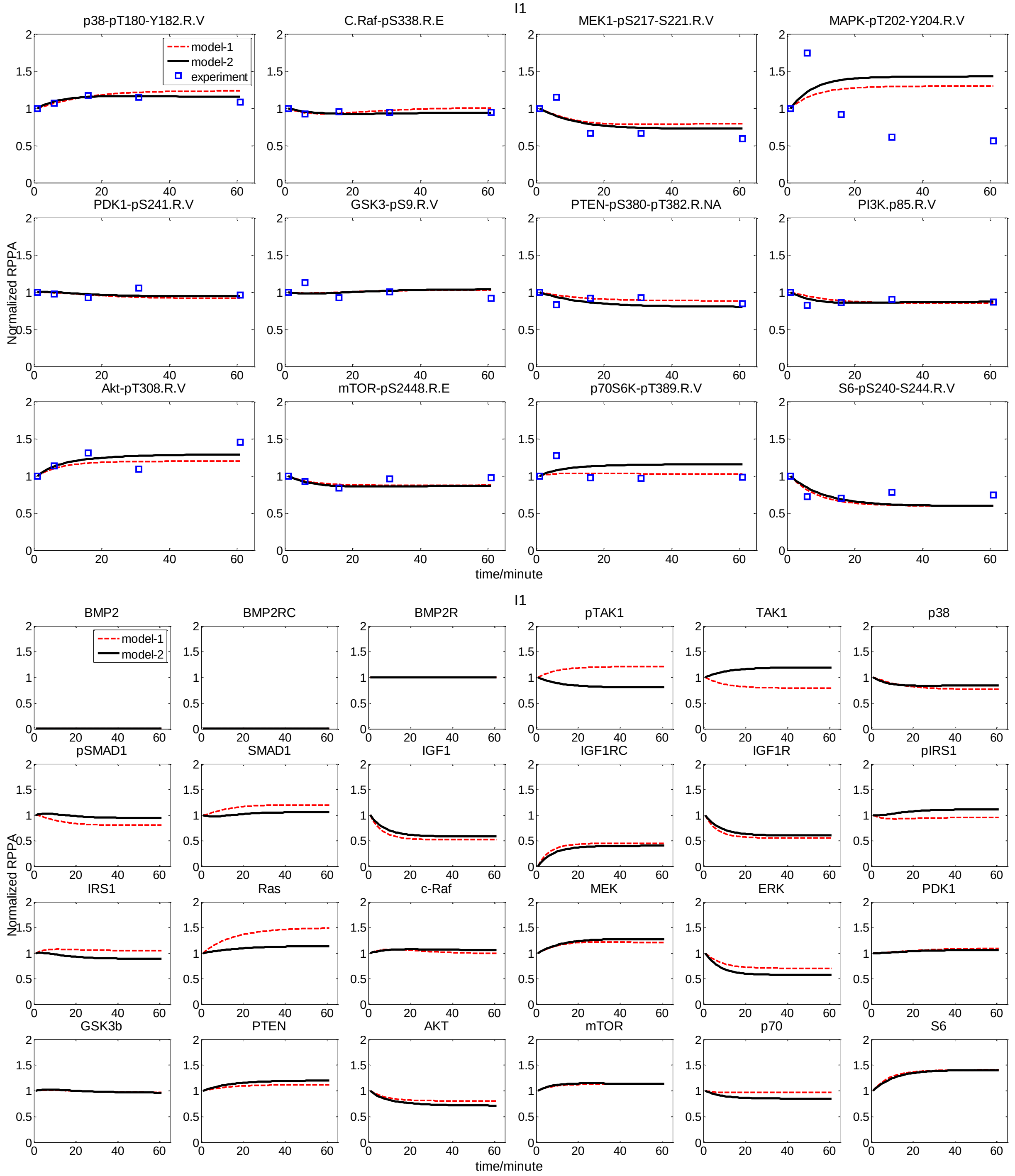

Figure S3. Fitting and prediction results of protein dynamics for treatment I1 (IGF-1 at day 1). Shown are data fitting results for 12 proteins for which RPPA data is available (upper panel) and prediction results for 24 molecules where RPPA data is unavailable (lower panel). The y-axis represents protein expression level measured by RPPA (supplementary experimental methods) and normalized to initial time of cytokine treatment. model-1: fitting on 7 time points; model-2: fitting on 5 time points.

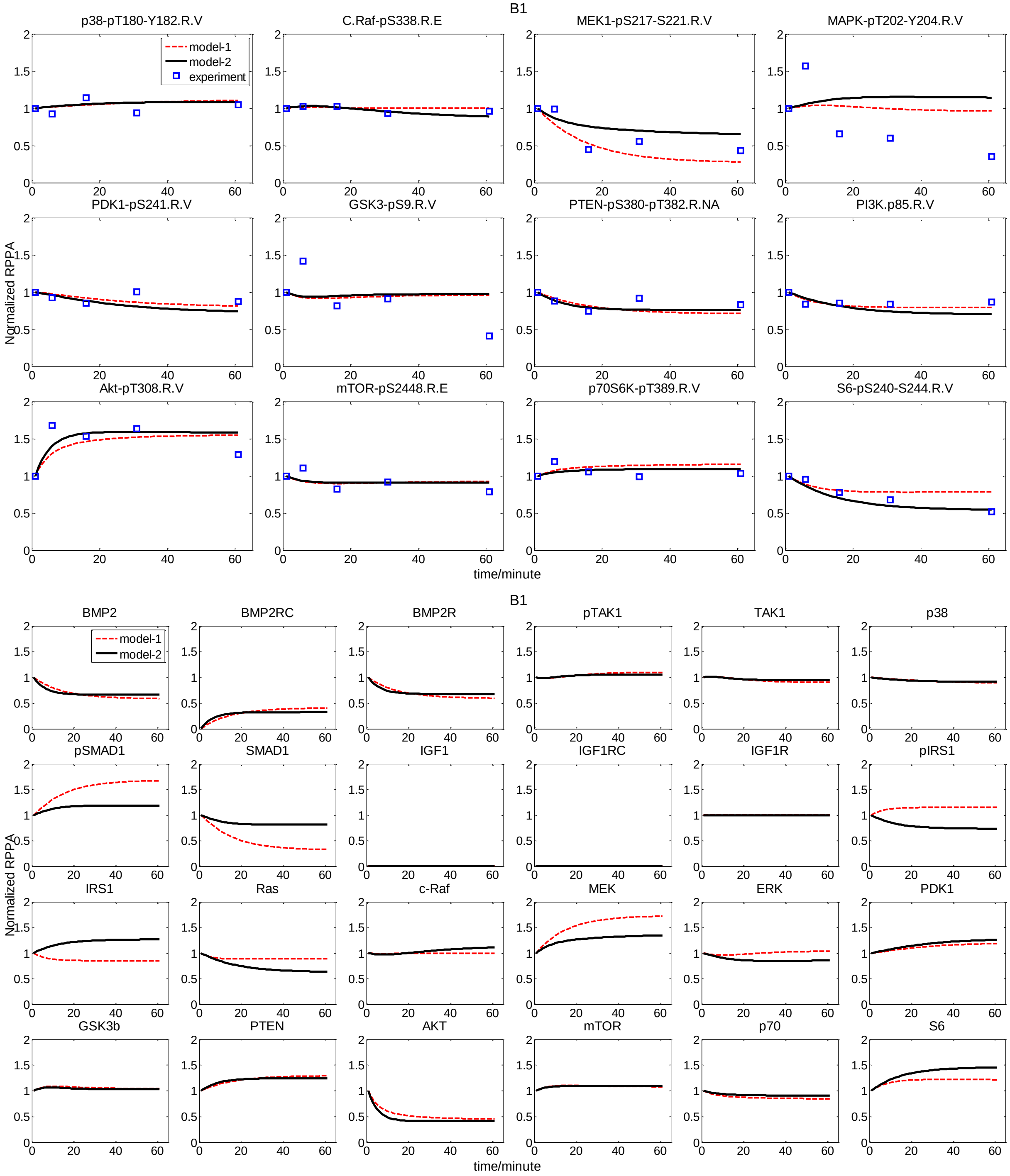

Figure S4. Fitting and prediction results of protein dynamics for treatment B1 (BMP-2 at day 1). Shown are data fitting results for 12 proteins for which RPPA data is available (upper panel) and prediction results for 24 molecules where RPPA data is unavailable (lower panel). The y-axis represents protein expression level measured by RPPA (supplementary experimental methods) and normalized to initial time of cytokine treatment. model-1: fitting on 7 time points; model-2: fitting on 5 time points.

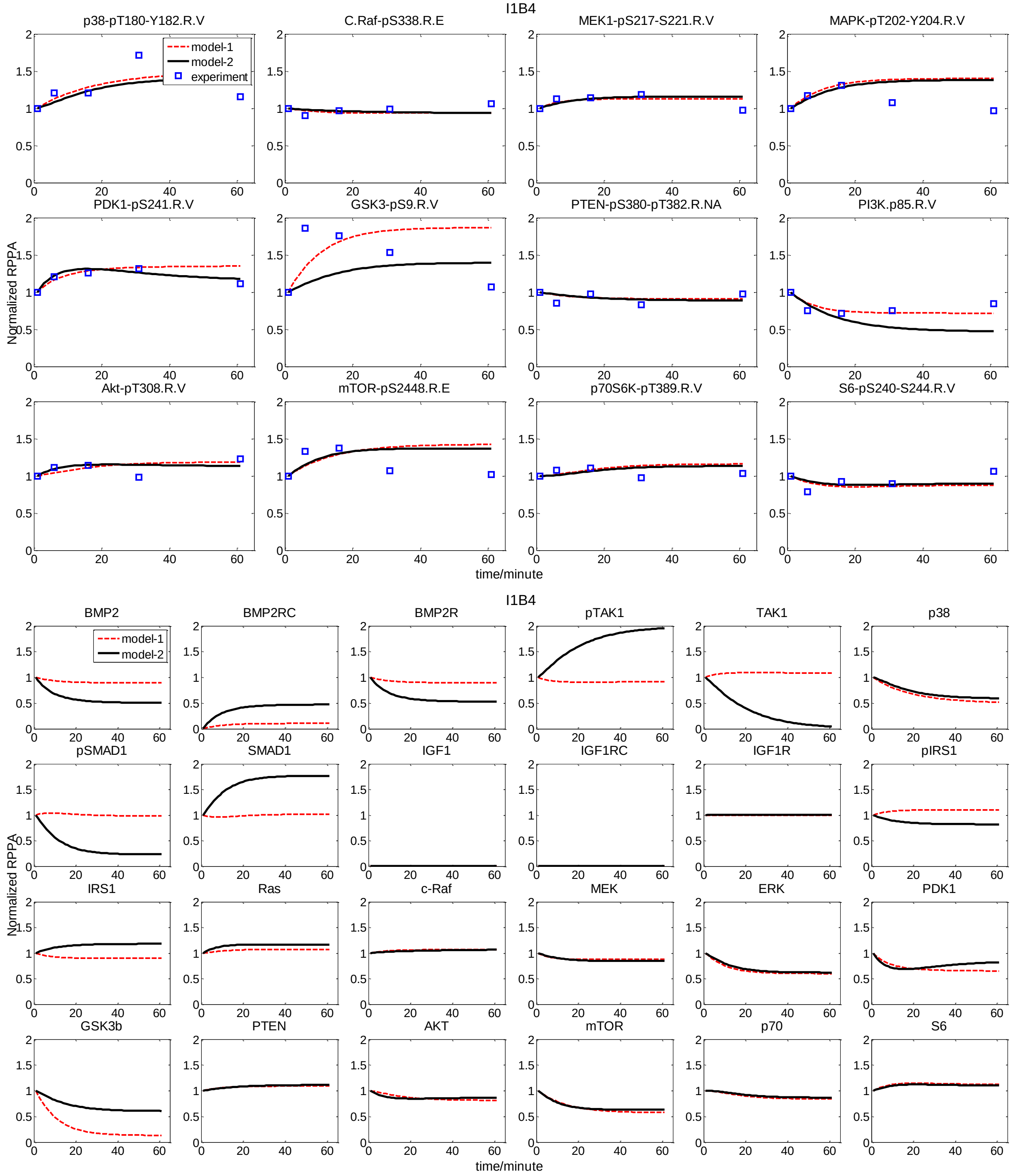

Figure S5. Fitting and prediction results of protein dynamics for treatment I1B4 (IGF-1 at day 1 followed by BMP-2 at day 4). Shown are data fitting results for 12 proteins for which RPPA data is available (upper panel) and prediction results for 24 molecules where RPPA data is unavailable (lower panel). The y-axis represents protein expression level measured by RPPA (supplementary experimental methods) and normalized to initial time of cytokine treatment. model-1: fitting on 7 time points; model-2: fitting on 5 time points.

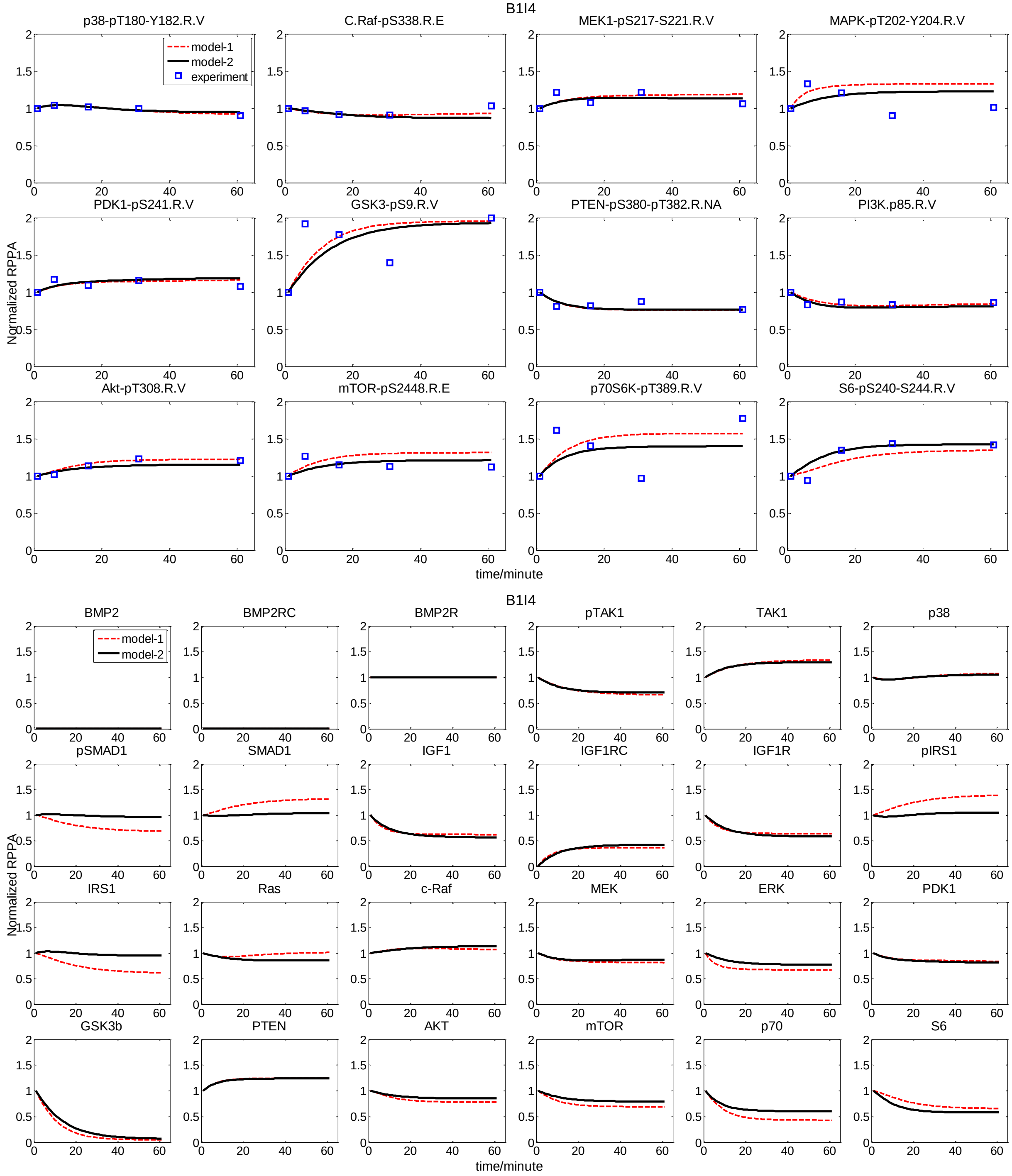

Figure S6. Fitting and prediction results of protein dynamics for treatment B1I4 (BMP-2 at day 1 followed by IGF-1 at day 4). Shown are data fitting results for 12 proteins for which RPPA data is available (upper panel) and prediction results for 24 molecules where RPPA data is unavailable (lower panel). The y-axis represents protein expression level measured by RPPA (supplementary experimental methods) and normalized to initial time of cytokine treatment. model-1: fitting on 7 time points; model-2: fitting on 5 time points.

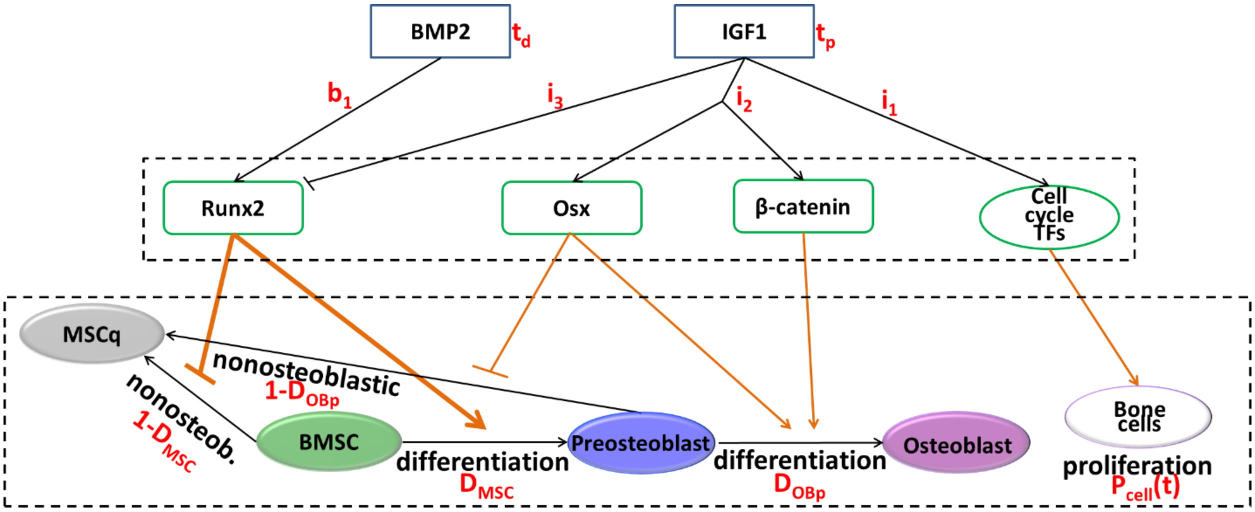

Figure S7. Compartmental model for the cellular lineage commitment with dynamic transfer rates. BMSC: bone marrow stromal cell; MSCq: non-osteoblastic cell. A detailed description of the related parameters are provided in Table S3.

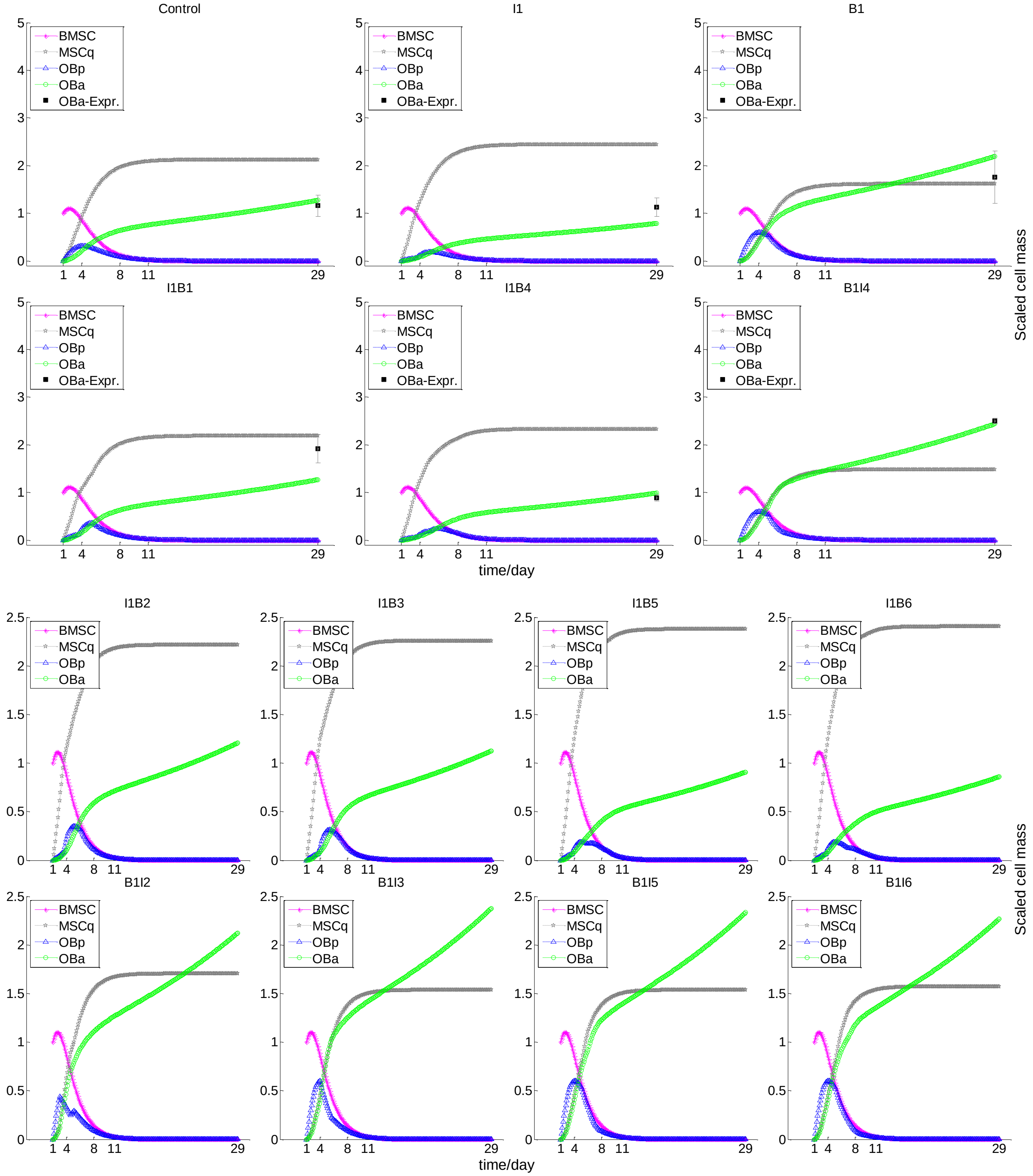

Figure S8. Experimental and model prediction results of cellular lineage dynamics under various cytokine combination treatments. The cellular composition (proportion of each cell type) displayed a wide heterogeneity, and distinct osteoblast formation was observed at day 29. Solid squares in the upper panel represent experimental data of osteoblasts (measured by ARS, see text). The lower panel represents cellular dynamics under various temporal combinations of cytokine treatments predicted by the model. BMSC: bone marrow stromal cell; MSCq: non-osteoblastic cells; OBp: preosteoblast; OBa: osteoblast; OBa-Expr.: experimental data for osteoblast formation measured by ARS.

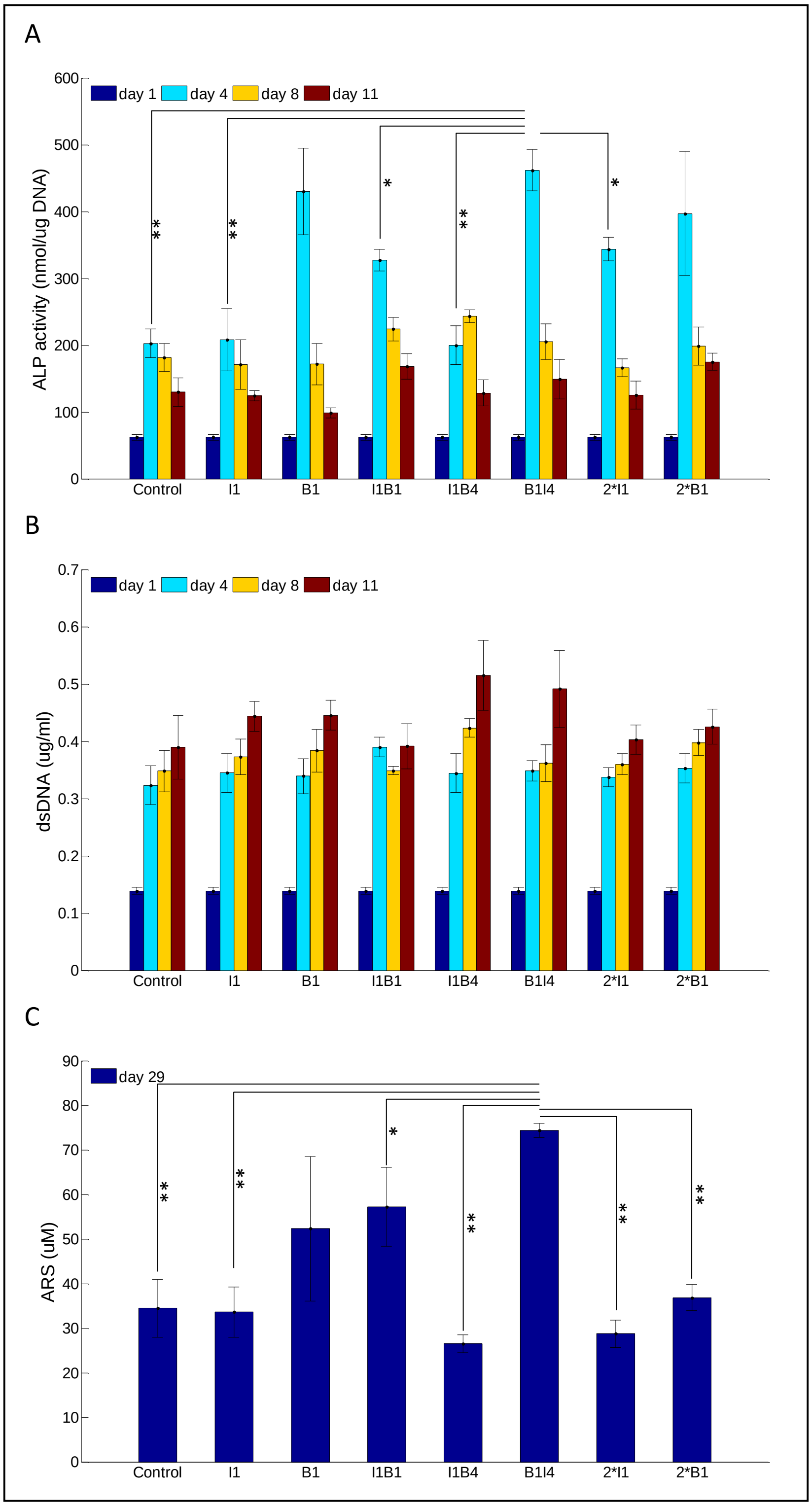

Figure S9. Experimental results for ALP (Alkaline Phosphate) activity (A), dsDNA (double stranded DNA) mass (B) and ARS (Alizarin Red S) staining (C) under different treatment conditions in W-20-17 cells. ALP is an early marker of osteoblastic differentiation of BMSCs; dsDNA reflects the total cell mass; while ARS measures the mineralization level reflective of osteoblast formation. More details are presented in the main text. The first 6 treatments refer to temporal combinations as shown in Table S1 at the concentration of 50ng/ml (Materials and Methods), while 2*I1 and 2*B1 correspond to doubled doses of respective single cytokines with 100ng/ml. ALP and dsDNA was measured at day 1, 4, 8, 11, and ARS was assayed at day 29. Data are presented as mean ± SD (n=4), *P<0.05 and **P<0.001 with two-tailed Student's *t*-test.

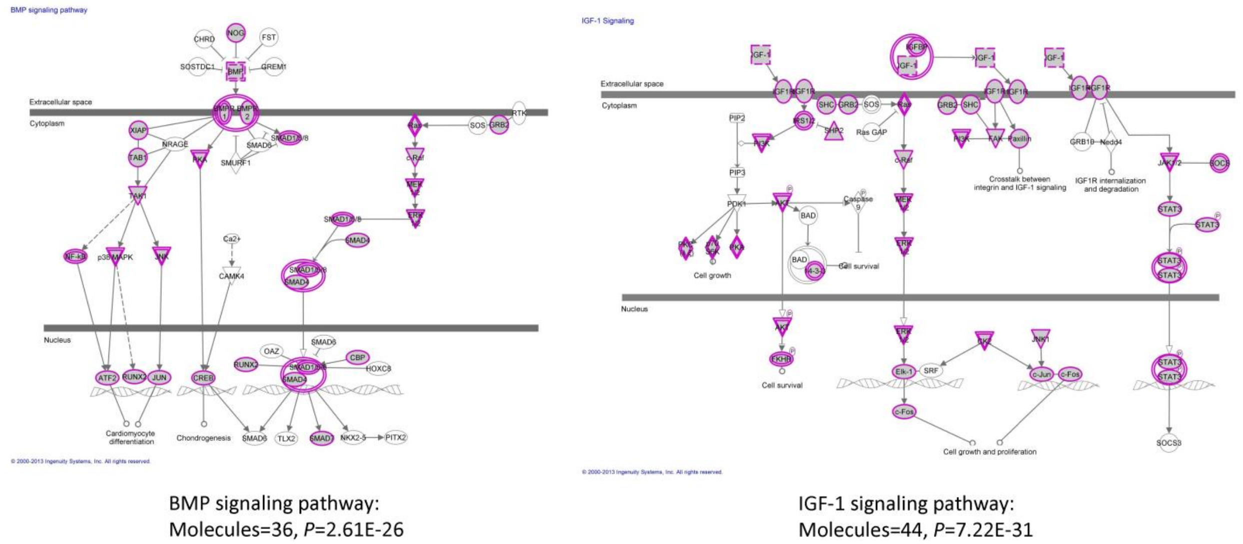

Figure S10. Canonical pathway mapping in IPA of interested biological molecules, including differentially expressed genes (DEGs) from microarray data, differentially activated proteins (DAPs) from reverse phase protein array data, and their possible upstream regulators (UTRs) collected using IPA.

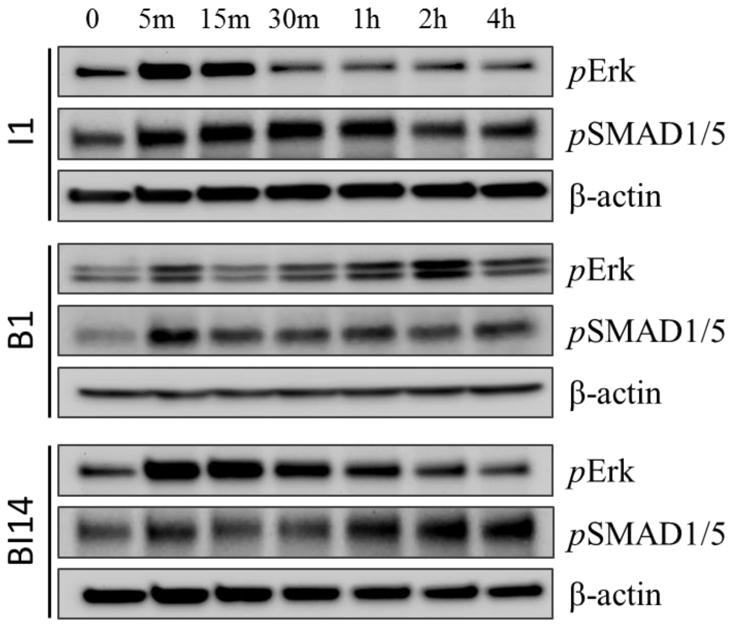

Figure S11. (Related to Figure 5) Experimental validation of critical molecular interactions and cytokine functions in MC-3T3 cells. MC-3T3 cells were treated with IGF-1 (I1) or BMP-2 (B1) or combined BMP-2 and IGF-1 (B1I4) as indicated (Table S1). The protein levels of pERk, pSMAD and β-actin (loading control) were determined by western blot at indicated time points. These results showed that in MC-3T3 cells, IGF-1 activated downstream ERK signals while BMP-2 triggered SMAD1/5, and ERK inhibited SMAD1/5 in various treatments especially in B1I4. These results are consistent with those obtained in W-20-17 cells.

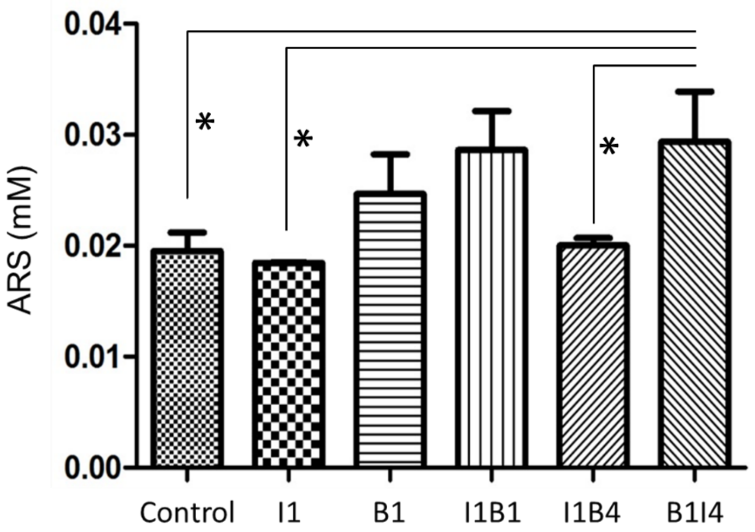

Figure S12. Experimental results for osteoblast formation (measured by Alizarin Red S staining at day 29 post-treatment) under different treatment scenarios in MC-3T3 cells. The B1, I1B1 and B1I4 groups displayed significantly more calcium deposition than those of the control, I1 and I1B4 groups. And the B1I4 group yielded greatest ARS intensity among all treatment conditions in MC-3T3 cells. Data are presented as mean ± SD (n=3), *P<0.05 with one-tailed Student's *t*-test.

Supplementary references

[1] Greenblatt MB, Shim JH, Zou WG, Sitara D, Schweitzer M, Hu D, et al. The p38 MAPK pathway is essential for skeletogenesis and bone homeostasis in mice. J Clin Invest. 2010;120:2457-73.
[2] Chen GQ, Deng CX, Li YP. TGF-beta and BMP Signaling in Osteoblast Differentiation and Bone Formation. Int J Biol Sci. 2012;8:272-88.
[3] Lee KS, Hong SH, Bae SC. Both the Smad and p38 MAPK pathways play a crucial role in Runx2 expression following induction by transforming growth factor-beta and bone morphogenetic protein. Oncogene. 2002;21:7156-63.
[4] Niba ETE, Nagaya H, Kanno T, Tsuchiya A, Gotoh A, Tabata C, et al. Crosstalk between PI3 Kinase/PDK1/Akt/Rac1 and Ras/Raf/MEK/ERK Pathways Downstream PDGF Receptor. Cell Physiol Biochem. 2013;31:905-13.
[5] Chappell WH, Steelman LS, Long JM, Kempf RC, Abrams SL, Franklin RA, et al. Ras/Raf/MEK/ERK and PI3K/PTEN/Akt/mTOR Inhibitors: Rationale and Importance to Inhibiting These Pathways in Human Health. Oncotarget. 2011;2:135-64.
[6] Kretzschmar M, Doody J, Massague J. Opposing BMP and EGF signalling pathways converge on the TGF-beta family mediator Smad1. Nature. 1997;389:618-22.
[7] Celil AB, Campbell PG. BMP-2 and insulin-like growth factor-I mediate osterix (Osx) expression in human mesenchymal stem cells via the MAPK and protein kinase D signaling pathways. Journal of Biological Chemistry. 2005;280:31353-9.
[8] Day TF, Guo XZ, Garrett-Beal L, Yang YZ. Wnt/beta-catenin signaling in mesenchymal progenitors controls osteoblast and chondrocyte differentiation during vertebrate skeletogenesis. Dev Cell. 2005;8:739-50.
[9] Ruvinsky I, Sharon N, Lerer T, Cohen H, Stolovich-Rain M, Nir T, et al. Ribosomal protein S6 phosphorylation is a determinant of cell size and glucose homeostasis. Gene Dev. 2005;19:2199-211.
[10] Chambard JC, Lefloch R, Pouyssegur J, Lenormand P. ERK implication in cell cycle regulation. Bba-Mol Cell Res. 2007;1773:1299-310.
[11] Shao HW, Peng T, Ji ZW, Su J, Zhou XB. Systematically Studying Kinase Inhibitor Induced Signaling Network Signatures by Integrating Both Therapeutic and Side Effects. Plos One. 2013;8.
[12] Peng H, Peng T, Wen J, Engler DA, Matsunami RK, Su J, et al. Characterization of p38 MAPK Isoforms for Drug Resistance Study Using Systems Biology Approach. Bioinformatics. 2014;30:1899-907.
